# Effects of Genetic Variation on Endurance Performance, Muscle Strength, and Injury Susceptibility in Sports: A Systematic Review

**DOI:** 10.3389/fphys.2021.694411

**Published:** 2021-07-21

**Authors:** Milena Appel, Karen Zentgraf, Karsten Krüger, Katharina Alack

**Affiliations:** ^1^Department of Exercise Physiology and Sports Therapy, Institute of Sports Science, Justus-Liebig-University Giessen, Giessen, Germany; ^2^Department of Exercise and Movement Science, Institute of Sports Sciences, Goethe-University Frankfurt, Frankfurt, Germany

**Keywords:** nucleotide polymorphism, genetic predisposition, endurance performance, injury susceptibility, muscle strength

## Abstract

The aim of this systematic review was to assess the effects of genetic variations and polymorphisms on endurance performance, muscle strength and injury susceptibility in competitive sports. The electronic databases PubMed and Web of Science were searched for eligible studies. The study quality was assessed using the RoBANS tool. Studies were included if they met the following criteria: (1) human study in English or German; (2) published in the period 2015–2019; (3) investigation of an association between genetic variants and endurance performance and/or muscle strength and/or endurance/strength training status as well as ligament, tendon, or muscle injuries; (4) participants aged 18–60 years and national or international competition participation; (5) comparison with a control group. Nineteen studies and one replication study were identified. Results revealed that the IGF-1R 275124 A>C rs1464430 polymorphism was overrepresented in endurance trained athletes. Further, genotypes of PPARGC1A polymorphism correlated with performance in endurance exercise capacity tests in athletes. Moreover, the RR genotype of ACTN3 R577X polymorphism, the C allele of IGF-1R polymorphism and the gene variant FTO T>A rs9939609 and/or their AA genotype were linked to muscle strength. In addition, gene variants of MCT1 (T1470A rs1049434) and ACVR1B (rs2854464) were also positively associated with strength athletes. Among others, the gene variants of the MMP group (rs591058 and rs679620) as well as the polymorphism COL5A1 rs13946 were associated with susceptibility to injuries of competitive athletes. Based on the identified gene variants, individualized training programs for injury prevention and optimization of athletic performance could be created for competitive athletes using gene profiling techniques.

## Introduction

Athletic performance and exercise-related injuries are multifactorial events resulting from extrinsic environmental factors and intrinsic factors such as genetic predisposition (Collins, 2009; Guilherme et al., 2014). The use of gene profiling techniques could be profitable to individually optimize training contents and to positively influence athletic performance (Pruna et al., 2016). For example, specific preventive training could be used to avoid muscular injuries in the presence of unfavorable genetic predisposition. Gene products modulate several physiological functions affecting performance and susceptibility to injuries in sports. For example, genes influence factors such as muscle fiber composition or the activity of aerobic and anaerobic enzymes. Moreover, genes and polymorphisms also predispose muscle strength or flexibility and the metabolic energy supply of an athlete (Guilherme et al., 2014). Therefore, a key role in athletic performance can be ascribed to these genetic factors, as their effectiveness can play a crucial role, especially in the elite sector (Posthumus and Collins, 2016). The present systematic review aims to evaluate currently presented genetic polymorphisms and their effects on top sporting performance by addressing endurance performance, muscle strength, and injury susceptibility.

Several genetic polymorphisms related to athletic performance have been detected (Ahmetov et al., 2016). The genetic contribution to performance-related factors can be determined by quantitative traits (Guilherme et al., 2014). These include, for example, the muscle fiber type distribution or muscle strength, but also outcome parameters of performance, such as running time. The focus of the current work was placed on the performance-determining factors endurance, muscle strength and injury susceptibility, as there is broad evidence that genetic predisposition has a high influence on these factors. The more the athletic performance is reflected in a complex interplay of variables such as environmental factors, technical abilities, psychological, sensorimotor or tactical skills, the less a clear genotype-phenotype relationship can be established. Additionally, novel research suggests that artificial factors such as transcranial alternating current stimulation (tACS) do not affect the explosive power considering the genetic profile of athletes (Giustiniani et al., 2021). Hence, performance in many team sports disciplines can only be associated with genetic variants to a limited extent. In contrast, genetic predisposition has a stronger influence on performance in sports disciplines in which the discipline-specific requirements are dominated by selected conditional performance factors, such as muscle strength or endurance capacity. In the following subchapters, the most important associations between endurance performance, muscle strength, and susceptibility to injury with genetic variations are identified and discussed.

Genetic variants associated with endurance performance have been studied the most to date. Endurance exercise performance refers to the resistance of the organism to fatigue and the rapid ability to regenerate after an exercise load. Based on the type of energy supply, aerobic and anaerobic endurance can be distinguished. For example, endurance events include long-distance runnings such as the Boston marathon or high-intensity interval runs (Spurway, 1992). Previous studies suggest that gene variants of ACE, PPAR, NRF 2, HIF-1 and VEGF are linked to endurance exercise performance capacity (Montgomery et al., 1998; Akhmetov et al., 2007; He et al., 2007; Eynon et al., 2009b; Döring et al., 2010; McPhee et al., 2011; Pokrywka et al., 2013). Most of these genes and transcription factors are related to enzymes of energy metabolism, play a role in the regulation of the cardiovascular system and are responsible for the storage of metabolites in muscles (Ahmetov et al., 2016). The gene variants of the ACE gene are frequently found in the literature. The ACE is part of the renin-angiotensin system and plays an important role in blood pressure regulation and maintenance of the water-electrolyte balance (Löffler and Petrides, 2013). Insertion of the DNA sequence of ACE is associated with increased endurance performance. The presence of the additional nucleotide fragment results in a lower activity of ACE in serum and tissue (Rigat et al., 1990; Danser et al., 1995). Accordingly, significantly improved muscle endurance (Montgomery et al., 1998) and positive effects on cardiorespiratory function are suspected (Hagberg et al., 1998). A link between the type II genotype and endurance performance was found in small, homogeneous, endurance-associated cohorts of athletes (Danser et al., 1995; Hagberg et al., 1998). However, so far these results could not be confirmed in other studies. The genes of the PPAR group also play an important role in the regulation of energy metabolism via mitochondria. Transcription factors of the PPAR group influence several genes involved in fat and carbohydrate metabolism and uptake of glucose into skeletal muscles (Löffler and Petrides, 2013). For example, Akhmetov et al. (2007) describe a significant overrepresentation of the C allele of the peroxisome proliferator-activated receptor delta (PPARD) +294 T>C in endurance athletes. In addition to genes of ACE and the PPAR group, polymorphisms of NRF-2 and HIF-1 have been shown to be associated with the endurance performance of athletes (McPhee et al., 2011; Pokrywka et al., 2013). Athletes who are carriers of these genetic polymorphisms have responded better to specific endurance training (Eynon et al., 2009b; McPhee et al., 2011).

The ability of skeletal muscles to generate strength and high contraction speeds is also affected by genetic factors (Eynon et al., 2013). In this context, the general term muscle strength refers to athletic performance. Muscle strength exercises include short, explosive, and fast powerful movements such as sprinting and throwing as well as series of power movements over a longer period. For example, the 100m-sprinting performance at the Olympic games is determined by muscle strength to a large extent. Muscle strength is largely determined by physiological factors such as muscle fiber size, type, length, and contraction speed. In particular, the distribution of muscle fiber types in favor of type 2 fibers can have a positive effect on an athlete's strength development (Tittel, 2016). Novel research suggests that other factors such as transcranial alternating current stimulation (tACS) does not affect the explosive power considering the genetic profile of athletes. To date, the polymorphism of the ACTN3 gene has been best investigated. This gene encodes for the protein ACTN3, which connects the structural elements within the skeletal muscle and thus participates in muscle contraction (Löffler and Petrides, 2013). ACTN3 is found exclusively in type 2 fibers (North et al., 1999). The R allele or RR genotype of the ACTN3 polymorphism is associated with strength or sprint performance of athletes. For example, Yang et al. (2003) demonstrated a significant overrepresentation of the R allele in elite sprinters as opposed to controls. Further studies confirm these results (Eynon et al., 2009a; Ahmetov et al., 2011). The evidence is supported by the fact that Yang et al. (2003) additionally found an association between the XX genotype of ACTN3 polymorphism and low sprint ability and muscle strength. However, to which extent this genotype is related to the performance of endurance athletes is controversial and the study situation is partially inconsistent regarding ACTN3 (Lucia et al., 2006). Furthermore, a variant of the ACE gene is discussed in connection with muscle strength: Wang et al. (2008) found an increased occurrence of the DD and ID genotype of the ACE gene in strength athletes and sprinters. The enzyme creatine kinase is also involved in the contraction of skeletal muscles. It transfers a phosphate group to the adenosine diphosphate consumed by muscle performance and is thus responsible for adenosine triphosphate resynthesis (Löffler and Petrides, 2013). The exact effects of gene variants of creatine kinase or its isoforms are not sufficiently understood (Pokrywka et al., 2013). However, they are assumed to be associated with reduced muscle fatigue (Macarthur and North, 2005). In contrast, a polymorphism of the myosin light chain kinase causes a greater loss of strength after exercise training (Clarkson et al., 2005). Finally, the gene polymorphisms of IGF-1 also play a role in the context of muscle strength (Lippi et al., 2010; Pokrywka et al., 2013; Ahmetov et al., 2016). In those cases, however, further scientific evidence is needed.

According to Guth and Roth the ability to be resistant to or recover from injuries is an important factor for optimal athletic performance (Guth and Roth, 2013). In addition to concussions and fractures, soft-tissue injuries in athletes are discussed in the literature in connection with polymorphisms and are also a focus of this review (Guth and Roth, 2013; Maffulli et al., 2013; Pokrywka et al., 2013). The most common evidence to date related to muscle, tendon and ligament injuries includes polymorphisms of COL1A1, COL5A1, MMP, and TNC (Pokrywka et al., 2013; Kaynak et al., 2017). COL1A1 and COL5A1 are structural elements of tendons and ligaments, the MMPs and TNC play a role in the interaction of tendons and the extracellular matrix (Löffler and Petrides, 2013). Their gene variants are thought to be associated with, among other things, anterior cruciate ligament rupture, tendinopathies, and achilles tendon rupture (Pokrywka et al., 2013; Kaynak et al., 2017; Czarnik-Kwaśniak et al., 2019). A study by Posthumus et al. (2009) compared athletes with anterior cruciate ligament rupture and healthy controls with respect to the distribution of a polymorphism of the COL5A1 (rs12722) gene and was able to demonstrate a gender-specific frequency to the detriment of the female athlete group. Stepien-Słodkowska et al. (2013) found that carriers of the G allele of the COL1A1 polymorphism have a reduced risk of cruciate ligament rupture compared to carriers of the T allele of the same polymorphism. Recently, further gene candidates and polymorphisms have been investigated, but these have not yet been fully understood. For example, the growth/differentiation factor 5 and the transforming growth factor beta 1 or their variants can also be associated with the above-mentioned injuries (Posthumus et al., 2010).

Summarizing the current state of evidence several genetic polymorphisms of performance-related genes such as ACE, NRF2, ACTN3, and COL are suggested to be expressed differently in elite athletes compared to non-athlete controls. However, the study design of existing exercise-related studies investigating genetic associations is very inconsistent. Therefore, this systematic review aims to minimize the effects of confounding factors such as origin, age, gender, or heterogeneous groups of athletes by defining strict inclusion and exclusion criteria to enable a comparison of the study results.

## Materials and Methods

### Search Strategy

This systematic review follows the PRISMA guidelines (Moher et al., 2009). In the period October to mid-December 2019, the two databases PubMed and Web of Science were searched for suitable studies on the topic “Effects of genetic variation on endurance performance, muscle strength and susceptibility to injury in sports.” The last search date was December 12, 2019. For the search on both databases thematically matching keywords and their synonyms were used. Preliminary search had shown that in most studies, endurance performance and muscle strength were investigated together, so they were combined into one category in the final search strategy. Table 1 gives an overview of the used headings, search terms and their synonyms. The detailed search strategy including topic, searched databases, search period and exact search term can be found in the Supplementary Table 1.

**Table 1:** Search strategy & search history.

**Heading**	**Search terms and synonyms**
**Topic:** genetic polymorphism for endurance & power, **Database:** PubMed, Web of Science; **Period:** 01.10. – 12.12.2019 **Search Terms:** athlete* OR professional player* OR professional athlete* OR elite athlete status* OR athletic status* OR competitive player* OR elite professional player* OR top-level athlete* OR top level athlete* OR competitive athlete* AND genetic variant* OR sports relevant polymorphism* OR genetic influence* OR genetic biomarker* OR genetic marker* OR polymorphism* OR single nucleotide polymorphism* OR genetic polymorphism* AND endurance capacity* OR endurance performance* OR endurance exercise* OR endurance* OR physical strength* OR power performance* OR power output* OR power sports performance* OR muscle power* OR muscle strength* OR power exercise* AND susceptibility to injury* OR muscle stiffness* OR soft tissue injury* OR tendinopathy* OR injury risk* OR ligament rupture* OR muscle injury* OR muscle strain injury* OR muscle damage* OR musculoskeletal soft tissue injury* OR ligament injury* OR tendon injury* OR injury*	
Athlete	Athlete, professional player, professional athlete, elite athlete status, athletic status, competitive player, elite professional player, top-level athlete, top level athlete, competitive athlete
Genetic polymorphism	Genetic variant, sports relevant polymorphism, genetic influence, genetic biomarker, genetic marker, polymorphism, single nucleotide polymorphism, genetic polymorphism
Endurance	Endurance capacity, endurance performance, endurance exercise, endurance
Power	Power performance, power output, power sports performance, muscle power, muscle strength, power exercise
Susceptibility to injury	Susceptibility to injury, muscle stiffness, soft tissue injury, tendinopathy, injury risk, ligament rupture, muscle injury, muscle strain injury, muscle damage, musculoskeletal soft tissue injury, ligament injury, tendon injury, injury

*The table gives an overview of the headings, search terms and their synonyms. The individual search terms were combined with the operator “OR,” the different levels/combined search terms with the operator “AND.”*

### Eligibility Criteria

To be included in this systematic review, studies had to meet each of the inclusion and none of the exclusion criteria (Table 2). The following criteria apply to all included studies:

(1) original study written in English or German,
(2) human study to ensure the transferability of the results,
(3) full-text studies published between 2015 and 2019,
(4) studies comparing a case group with a control group.

**Table 2:** Eligibility criteria.

**Criteria**	**Inclusion criteria**	**Exclusion criteria**
**General criteria**		
Type of study	Original study Human study	Review Meta-analysis Review article Narration Letter Book chapter Animal study *In vitro* study Twin study Family study
Language	Englisch, German	Other languages
Period	2015–2019	<2015 > 2019
Full Text	Full Text available	Full Text not available Abstracts
Control group	Control group	No control group
**Specific criteria according to the PICO-scheme**		
Population	Elite athletes, who take part in (inter-) national competitions Age: 18–60 years	Athletes, who take part in regional competitions Recreational athletes Amateur athletes Non-Athletes Groups of people with neurological, internal, or metabolic diseases <18 years > 60 years
Intervention	Genetic variants with influence on endurance performance, muscle strength and injury susceptibility (only soft-tissue injuries in form of muscle, tendon, or ligament damage)	Genetic variants with influence on other orthopedic diseases or injuries well as internal, metabolic or neurological or neuropsychological diseases
Comparison	Control group (athletes, non-athletes)	Groups of people with orthopedic, internal, metabolic, or neurological diseases/injuries
Outcome	Analysis or comparison of the investigated polymorphism with gene profiles of competitive athletes or association of a genetic polymorphism with physiological data or athletic performance	Justification of polymorphism with elite status or competition placements

*The table gives an overview of the general selection criteria and specific criteria according to the PICO scheme. For inclusion the studies had to meet each of the general and specific inclusion and none of the exclusion criteria*.

Studies were included investigating gene variants and polymorphisms in a study population of female and male athletes between 18 and 60 years of age who participate in national and/or international competitions or who were defined as “elite,” “sub-elite,” “professional,” or “top level” or could be classified as members of this group based on their performances. Regarding injury susceptibility, only soft-tissue injuries such as muscle, tendon or ligament damage were considered, as those are the most common sports injuries (Bauer, 2013) and have been best studied to date in relation to polymorphisms (Maffulli et al., 2013; Pokrywka et al., 2013). Accordingly, other orthopedic diseases or injuries, such as stress fractures or concussions, as well as internal, metabolic, or neurological or neuropsychological diseases that may be associated with gene variants, have not been considered. In addition, the studies should include at least an analysis or comparison of the genetic profiles of endurance and/or strength athletes or athletes with an injury and/or an association of this genetic profile with physiological data or the athletic performance of the groups. An exclusive association with competition placements or the elite status of athletes was not considered.

### Study Selection Process

**Figure 1:**
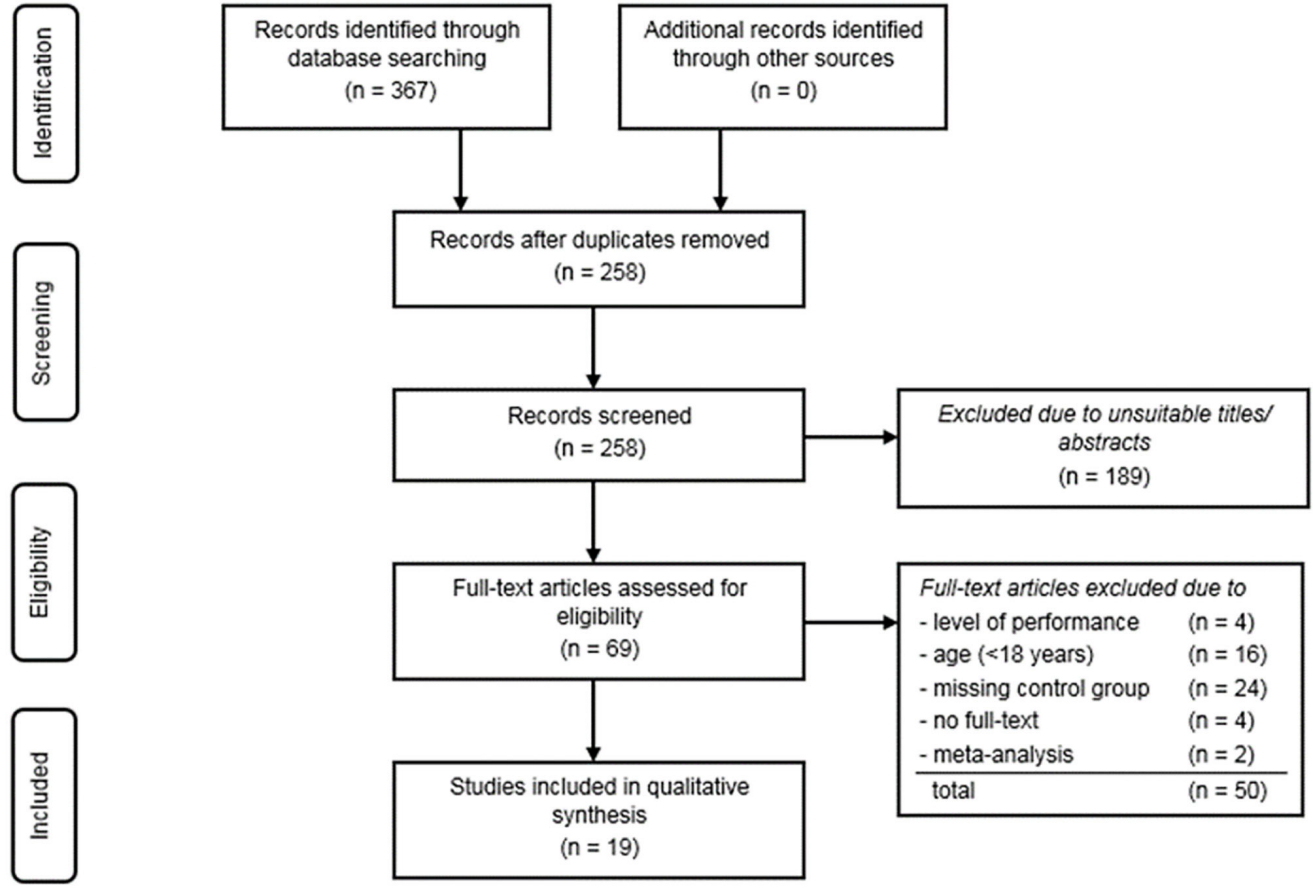
Flow chart of the study selection process.

The complete study selection process is presented by a flow chart in Figure 1. After removing the duplicates, all remaining articles were checked for meeting all inclusion and none of the exclusion criteria. The characteristics of the studies included are presented in Tables 4, 5. The risk of bias was assessed using the RoBANS tool (Kim et al., 2013) and following the approach of Kaynak et al. (2017). The following domains are addressed by this tool: selection of participants, consideration of possible confounders, exposure measurement, blinding and completeness of results, and reporting of results (Kim et al., 2013). The bias potential within a category can be assessed as “low” (↓), “high” (↑), or “ambiguous/unclear” (?). To ensure the greatest possible objectivity, the evaluation of the individual categories was based on the evaluation procedure of the ROB instrument (Kim et al., 2013). Like Kaynak et al. (2017), the RoBANS instrument was slightly modified for this review. Since all included studies had a cross-sectional or case-control design, the participant withdrawal category was not included in the analysis. Therefore, the exposure measurement category was divided into two subgroups: First, the definition of exposure and, second, the measurement of exposure. In the category “selection of participants,” a clear definition, a reasonable division into groups, an adequate selection of participants of the control group matching the case group, and homogeneity and representativeness of the case group were required. For assessing the selection bias due to confounding factors, gender, age, and origin were considered in the present systematic review. In this case, design methods or statistical measures such as matching, stratification or adjustment were intended to eliminate disruptive factors. In addition, the subdivision of athletes into specific groups seemed to be useful in studies that included both strength and endurance athletes. Blinding of the results and appropriate reporting were also important. The procedure for quality assessment is presented in the Supplementary Table 2.

## Results

### Selection of Studies

A total of 367 hits could be identified by screening the databases PubMed and Web of Science. After removing duplicates, 258 articles were included in the pre-selection category (Figure 1). By checking the titles and abstracts of the pre-selection, reviews, meta-analyses, review articles, animal studies and book chapters were excluded in the next step. Finally, 69 hits were checked for their suitability using the inclusion and exclusion criteria. A total of 24 studies were excluded due to a missing control group. Moreover, 20 studies were eliminated because of investigating adolescents (<18 years) or an insufficient performance level of the athletes. In addition, four of the studies were excluded due to a missing full-text version and two other studies were classified as meta-analyses. Finally, a total of 19 remaining articles could be identified and included for a qualitative assessment.

### Study Characteristics

The study characteristics of the 19 included studies are summarized in Tables 3, 4. The characteristics include study design, number and age of cases and controls, gender of the participants, population, or origin, genes studied and their polymorphisms. All athletes were at least at national competition level. Data not listed or not accessible to the user were marked with “not listed (N/A).” In total, 13 of the 19 studies dealt with genetic polymorphisms related to endurance performance and muscle strength, whereas six of the studies thematize susceptibility to injuries. The article by Zarebska et al. (2017) included an original and a replication study with two different independent groups of athletes. Therefore, the two studies have been considered separately. Not all 13 studies made explicit reference to their chosen study design. In the absence of information on the design, the review article by Guilherme et al. (2014) and its explanation of observational studies in genetic association studies was used to assess the design. Thus, five cross-sectional and nine case-control studies were finally identified. The 13 included references studied 17 different genes and 23 different polymorphisms related to endurance and muscle strength of competitive athletes. The variants of the following genes or their products were examined: ACE, ACTN3, ACVR1B, AGT, CNDP1, CNDP2, FTO, GSTP1, IGF-1R, MCT1, NFR-2, PPARD, PPARG, PPARGC1A, and TFAM. The respective polymorphisms of the mentioned genes are listed in Table 3. The studies related to endurance performance and muscle strength, mainly focused on genes whose enzymes, transcription factors and receptors are involved in energy metabolism. NRF-2, PPARD, PPARG, PPARGC1A, and TFAM, for example, regulate the mitochondrial metabolism (Kelly and Scarpulla, 2004) and CNDP1 or CNDP2, IGF-1R, and MCT1 are involved in muscular energy metabolism (Dubouchaud et al., 2000; Philippou et al., 2007; Harris et al., 2012). ACVR1B acts as a growth and differentiation factor in the muscle (Windelinckx et al., 2011), FTO influences the anthropometric requirements of an athlete via body mass (Speakman, 2015) and GSTP1 provides an antioxidant response to oxidative stress (Hayes et al., 2005). The enzyme ACE plays a role in the regulation of blood pressure and electrolyte and water balance and ACTN3 is integrated as a protein in muscle contraction (Löffler and Petrides, 2013). The other six studies focused on the vulnerability of athletes to injury. All authors stated that they had conducted their research in the form of a case-control design. A total of 30 different polymorphisms of 14 genes were investigated in connection with soft tissue injuries. In four studies a possible association of anterior cruciate ligament rupture with gene variants of the following genes or their products was discussed: IL1B, IL6, IL6R, MMP3, MMP8, TIMP2, COL5A1, and TNC. Furthermore, gene variants of FOXP3, FCRL3, BMP4, and FGF3 and FGF10 as well as FGFR1 were analyzed in connection with tendinopathy in competitive athletes. The respective polymorphisms are shown in Table 4. On the one hand, the functions of the listed genes and their protein products are associated with differentiation, regeneration, modulation, and regulation of the metabolism of ligaments and tendons (BMP4, COL5A1, FGFs, MMPs, TIMP2, TNC) (Somerville et al., 2003; Brent and Tabin, 2004; Chiquet-Ehrismann and Tucker, 2011; Löffler and Petrides, 2013). On the other hand, some of them have an immunological function such as genes for the regulation of the expression of interleukins and immune regulating proteins (IL1B, IL6, IL6R, FOXP3, FCRL3) (Löffler and Petrides, 2013).

**Table 3:** Characteristics of included studies on endurance and muscle strength.

**Author(s)/Year**	**Design**	**Number (n); age (MW ± SD) of cases**	**Number (n); age (MW ± SD) of controls**	**Gender**	**Population**	**Gene(s) (Polymorphism, Gene variant)**
Ben-Zaken et al. (2015)	Cross-sectional study	159; 35.9 ± 12.2	96; 26 ± 3	M, F	Israeli	IGF-1R (275124 A>C rs1464430)
Falahati and Arazi (2019)	Cross-sectional study	29; 38.5 ± 16.5	28; 38.9 ± 16.8	M	Iranian	ACE (I, D)
Ginszt et al. (2018)	Case-control study	100; 18–37 years	100; 23–44 years	M, F	Polish, Russian, Austrian	ACTN3 (R577X)
Guilherme et al. (2019)	Case-control study	677 (b); 28.5 ± 10.3 920 (r); 23.3 ± 4.3	652 (b); 32.3 ± 17.4 754 (r); 20.1 ± 2.6	M, F	Brazilian (b), Russian (r)	FTO (T>A rs9939609)
Guilherme and Lancha (2017)	Case-control study	908; 27.1 ± 7.7	967; 32.4 ± 12.0	M, F	Brazilian	CNDP1 (rs733686, rs2887) CNDP2 (rs12964619, rs6566810, rs3764509, rs734559, rs7577) CNDP1; CNDP2 (rs2346061)
Jin et al. (2016)	Cross-sectional study	111; 21.1 ± 2.26	145; 21.3 ± 2.54	M, F	Korean	PPARD (T294C rs2016520) PPARGC1A (Gly482Ser rs8192678)
Kikuchi et al. (2017)	Cross-sectional study	199; N/A	649; N/A	N/A	Japanese	MCT1 (T1470A rs1049434)
Li et al. (2017)	Case-control study	160; 20 ± 2	206; 20 ± 1	M, F	Chinese	ACTN3 (R577X)
Peplonska et al. (2017)	Case-control study	413; 23.5 ± 4.7	451; 23.0 ± 3.1	M, F	Polish	ACE (I, D rs4341) ACTN3 (R557X rs18157 39) AGT (M235T rs699) NRF-2 (rs12594956) PPARGC1A (G482S rs8192678) PPARG (P12A rs1801282) TFAM (S12T rs1937, rs2306604)
Voisin et al. (2016)	Case-control study	1,672; N/A	1,089; N/A	N/A	Brazilian, Italian, Polish, Russian	ACVR1B (rs2854464)
Yang et al. (2017)	Cross-sectional study	103; N/A	50; N/A	M, F	Chinese	ACTN3 (R577X rs1815739)
Zarebska et al. (2017)	Case-control study	507; 23.5 ± 0.4	562; 27.6 ± 3.2	M, F	Russian	GSTP1 (c.313 A>G)
Zarebska et al., 2017 (replication study)	Case-control study	510; 29.1 ± 6.3	684; 20.9 ± 2.1	M, F	Polish	GSTP1 (c.313 A>G)

*n, number; MW, mean value; SD, standard deviation; M, male; F, female; N/A, not specified*.

**Table 4:** Presentation of characteristics of included injury susceptibility studies.

**Author(s)/Year**	**Design**	**Number (n); age (MW ± SD) of participants**	**Number (n); age (MW ± SD) of controls**	**Gender**	**Population**	**Gene(s), (Polymorphism, Gene variant)**
Lulińska-Kuklik et al. (2019b)	Case-control study	229; 26 ± 4 (M), 25 ± 4 (F)	194; 25 ± 3 (M), 29 ± 2 (W)	M, F	Polish, Eastern European	IL1B (rs16944, rs1143627) IL6 (rs1800795) IL6R (rs2228145)
Lulińska-Kuklik et al. (2019c)	Case-control study	229; 26 ± 4 (M), 26 ± 6 (F)	192; 25 ± 3 (M), 29 ± 2 (W)	M, F	Polish, Eastern European	MMP3 (rs591058, rs679620) MMP8 (rs11225395) TIMP2 (rs4789932)
Lulińska-Kuklik et al. (2019a)	Case-control study	229; 26 ± 4 (M), 25 ± 4 (F)	192; 25 ± 3 (M), 29 ± 2 (W)	M, F	Polish, Eastern European	TNC (rs1330363, rs2104772, rs13321)
Lulińska-Kuklik et al. (2018)	Case-control study	134; 23.4 ± 3.1	211; 25.3 ± 3.4	M	Polish, Eastern European	COL5A1 (rs12722, rs13946)
Salles et al. (2018)	Case-control study	125; 26.86 ± 6.03	146; 21.62 ± 5.39	M, F	Brazilian	FOXP3 (−2,383 C>T rs3761549) FCRL3 (−169 T>C rs7528684)
Salles et al. (2015)	Case-control study	52; 30.23 ± 4.72	86; 27.33 ± 4.67	M	Brazilian	BMP4 (rs2761884, rs17563, rs2855529, rs2071047, rs762641) FGF3 (rs7932320, rs1893047, rs12574452, rs4631909, rs4980700) FGF10 (rs1448037, rs900379, rs1011814, rs593307) FGFR1 (rs13317)

*n, number; MW, mean value; SD, standard deviation; M, Male; F, Female*.

### Risk of Bias Within the Studies

**Figure 2:**
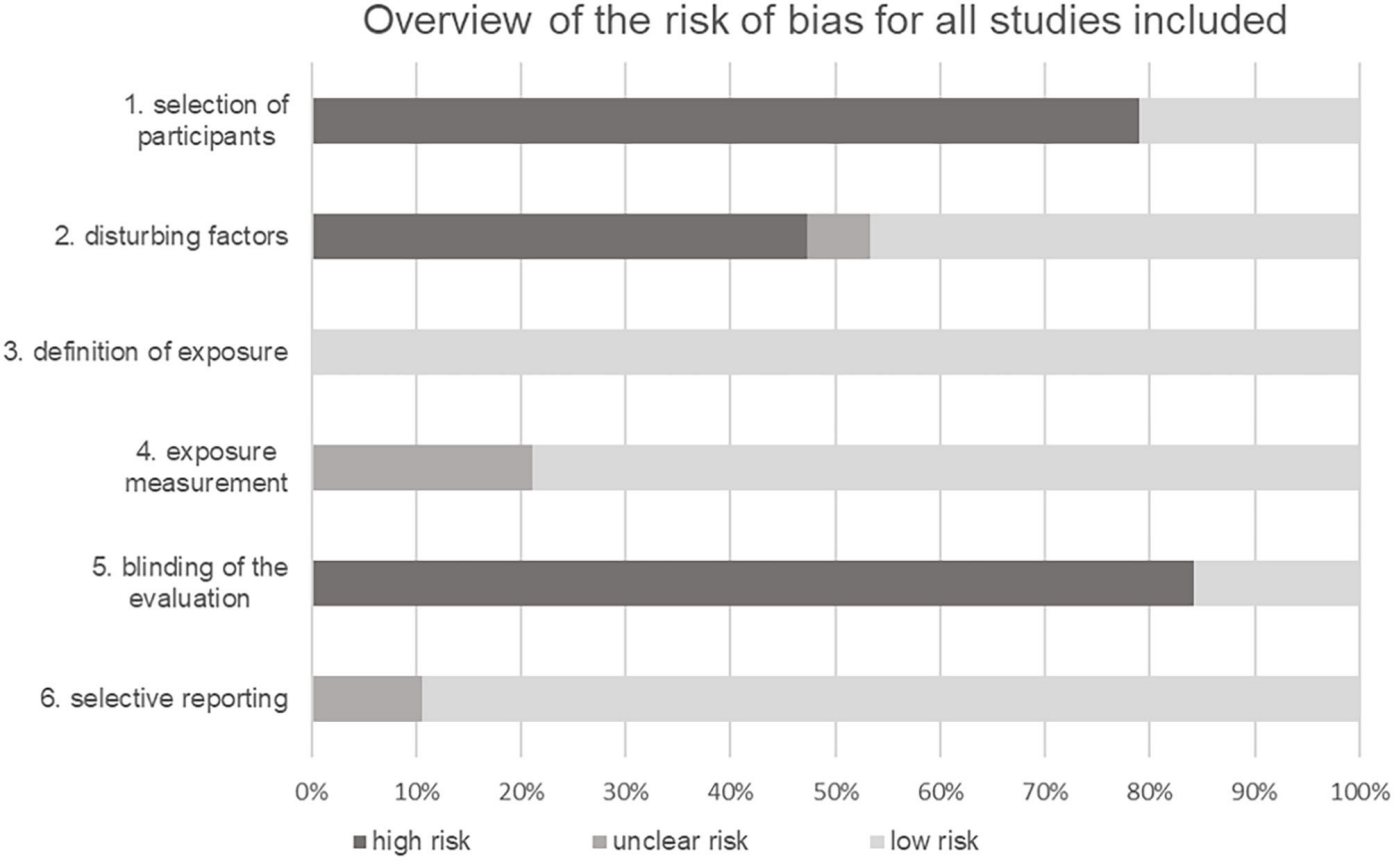
Overview of the risk of bias for all studies in the categories of quality assessment.

The RoBANS instrument was used to assess the quality of the studies (Kim et al., 2013). Since all studies were non-randomized observational studies, this instrument was considered useful for evaluation. The risk of bias for each study is shown in Table 5. An illustration graphically summarizes the results of the quality assessment of all studies in each category (Figure 2). In 79% of the included studies, a high risk of bias for the selection of participants could be found. In contrast, only 21% of the studies showed a low selection bias. Most authors dealt with possible confounding factors. In total, 47% of the studies used a suitable statistical methodology or design to counter possible confounders. Furthermore, 47% of the studies could not demonstrate adequate measures for considering the confounding factors age, gender, origin, and sports discipline. For one study the bias potential could not be clearly determined due to limited access. Furthermore, exposure was clearly defined in all studies (100%). In 79% of the studies, the measurement method for exposure was considered suitable. Only 21% of the references described their measurement method in little detail and with little information, which is why the bias potential in these cases could not be clearly assessed. No blinding of the investigators when evaluating their results was noted in 84% of the studies, only 16% indicated or described in detail an adequate blinding strategy. The selective reporting category included 89% of all studies, discussing in detail both significant and non-significant results. In three studies, further graphs and tables were referred to, but were not shown, which is why an unclear bias potential was noted for these 11%. None of the studies showed a high reporting risk.

**Table 5:** Summary of the quality assessment of all included studies on endurance performance and muscle strength as well as on injury susceptibility.

**Study**	**Selection of participants**	**Confounding factors**	**Definition of exposure**	**Exposure measurement**	**Blinding of the result evaluation**	**Selective reporting on results**
Ben-Zaken et al. (2015)	↑	↑	↓	↓	↑	↓
Falahati and Arazi (2019)	↑	↓	↓	↓	↓	↓
Ginszt et al. (2018)	↑	↑	↓	?	↑	↓
Guilherme et al. (2019)	↓	↓	↓	↓	↑	↓
Guilherme and Lancha (2017)	↑	↑	↓	↓	↑	↓
Jin et al. (2016)	↑	↑	↓	↓	↑	↓
Kikuchi et al. (2017)	↑	?	↓	↓	↑	?
Li et al. (2017)	↓	↓	↓	?	↑	↓
Peplonska et al. (2017)	↑	↓	↓	↓	↑	↓
Voisin et al. (2016)	↑	↑	↓	↓	↑	?
Yang et al. (2017)	↑	↓	↓	↓	↓	↓
Zarebska et al. (2017)	↓	↑	↓	?	↑	↓
Zarebska et al. (2017)	↑	↑	↓	↓	↑	↓
Lulińska-Kuklik et al. (2019a)	↑	↓	↓	↓	↑	↓
Lulińska-Kuklik et al. (2019b)	↑	↑	↓	↓	↑	↓
Lulińska-Kuklik et al. (2019c)	↑	↑	↓	↓	↑	↓
Lulińska-Kuklik et al. (2018)	↑	↓	↓	↓	↓	↓
Salles et al. (2018)	↓	↓	↓	↓	↑	↓
Salles et al. (2015)	↑	↓	↓	?	↑	↓

*↑, high risk of bias; ↓, low risk of bias; ?, unclear risk of bias*.

### Results of the Studies

The results of the individual studies are presented in Tables 6, 7.

**Table 6:** Results of included studies investigating an association between genetic variants and endurance and muscle strength.

**Author(s)/year**	**Gene(s)**	**Topic**	**Results [with specification p =, OR =, 95% KI (…), r =]**
Ben-Zaken et al. (2015)	IGF-1R	Regulation of IGF-1R^1^ polymorphism in ET and ST vs. CG	- Genotype and allele frequencies of ET and ST vs. KG n. s. - AA genotype ↑ in Et vs. ST (*p < * 0.05; r = 0.66) - C allele ↑ in ST vs. endurance athletes (*p < * 0.05; r = 0.66)
Falahati and Arazi (2019)	ACE	Effects of the genotypes of ACE^1^ on cardiovascular factors in male footballers vs. CG	- Genotype distribution of ACE (I/D) in athletes vs. KG n. s. - Effects of ACE (I, D) on VO_2_ max, blood pressure (systolic, diastolic), body fat and resting heart rate n. s.
Ginszt et al. (2018)	ACTN3	Detection of ACTN3^1^ genotypes in bouldering (B) and classical climbing (CC) vs. CG	- Genotype and allele frequencies in athletes (B + CC) vs. CG n. s. - Following classification of athletes in B and CC: → RR ↑, RX and XX ↓ in B vs. KK + CG (*p =* 0.0017) → R ↑, X ↓ in B vs. KK + CG (*p =* 0.0004) → Genotype and allele frequencies in KK vs. B + KG n. s.
Guilherme et al. (2019)	FTO	Analysis of FTO^1^ polymorphism in ST, ET, MT, and GS of two cohorts vs. CG	- Allele and genotype distribution of FTO polymorphism in Brazilian and Russian ET vs. CG n. s. - Following classification of athletes in medium (MDR) and long-distance runners (LDR): → AA genotype ↓ in Brazilian LDR vs. Brazilian MDR (*p =* 0.048); Brazilian LDR vs. CG n. s. → A allele ↓ in Russian LDR vs. Russian MDR (*p =* 0.014); Russian LDR vs. CG n. s. - FTO polymorphism in Brazilian ST vs. CG n. s. - A allele in dominant genotype distribution ↑ in Russian ST vs. CG (*p =* 0.009) - TA + AA genotype vs. TT ↑ in Russian ST vs. CG [*p =* 0.002; OR = 1.45; 95% CI (1.06–1.97)] - Allele and genotype distribution of FTO polymorphism in Russian GS vs. CG n. s. - Allele and genotype distribution of FTO polymorphism in Russian MT vs. CG n. s. - A allele ↑ in Brazilian MT vs. CG (*p =* 0.025) - TA ↑ in Brazilian MT vs. CG [*p =* 0.046; OR = 1.49; 95% CI (1.01–2.20)] - AA ↑ in Brazilian MT vs. CG [*p =* 0.042; OR = 1.70; 95% CI (1.02–2.84)] - Following classification of Russian and Brazilian MT into weight categories (heavy, light): → AA genotype (in the recessive model) ↑ in “heavy” Russian MT vs. CG (*p =* 0.025) → A allele ↑ in “heavy” Brazilian MT. vs. CG (*p =* 0.005) - Following combining groups: → A allele dominant ↑ in “heavy” MT vs. CG [*p =* 0.018; OR = 1.79; 95% CI (1.10–2.90)] → A allele recessive ↑ in “heavy” MT vs. CG [*p =* 0.015; OR = 1.91; 95% CI (1.13–3.22)]
Guilherme and Lancha (2017)	CNDP1 CNDP2	Analysis of polymorphisms of CNDP1^1^ and CNDP2^1^ in ST, ET, and, MT vs. CG	- Allele and genotype frequencies of polymorphisms in ET vs. CG n. s. - CNDP2 rs3764509 for ST vs. CG: → GG vs. CC genotype ↑ in ST vs. CG [*p =* 0.005; OR = 1.66; 95% CI (1.16–2.38)] → GC vs. CC genotype ↑ in ST vs. CG [*p =* 0.016; OR = 1.51; 95% CI (1.08–2.11)] → GG + CG vs. C/C genotype ↑ in ST vs. CG [*p =* 0.022; OR = 1.32; 95% CI (1.04–1.67)] → After adjustment: only GG genotype ↑ in ST vs. CG (*p < * 0.05) - CNDP1; CNDP2 rs2346061 for ST vs. CG: → AC genotype ↑ in ST vs. CG [*p =* 0.03; OR = 1.31; 95% CI (1.02–1.68)] → AC + CC vs. AA genotype ↑ in ST vs. CG [*p =* 0.015; OR = 1.34; 95% CI (1.05–1.69)] - CNDP1; CNDP2 rs2346061 for MT vs. CG → AC genotype ↑ in MT vs. CG [*p =* 0.015; OR = 1.54; 95% CI (1.08–2.19)] → AC + CC genotype ↑ in MT vs. CG [*p =* 0.018; OR = 1.50; 95% CI (1.07–2.10)] - CNDPD1 rs2887 distribution significant different in ST vs. MT (even after adjustment): → AA vs. GG genotype ↑ in ST vs. MT [*p =* 0.033, OR = 0.48; 95% CI (0.25–0.94)] → AA vs. GA + GG genotype ↑ in ST vs. MT [*p =* 0.36; OR = 0.51; 95% CI (0.27–0.95)]
Jin et al. (2016)	PPARD PPARGC1A	Detection of PPARD^1^ and PPARGC1A^1^ polymorphisms in athletes vs. CG + their relationship to the athletic performance shown in seven different performance tests	- Genotype and allele distribution for PPARD T294C, PPARGC1A Gly482Ser in athletes vs. CG n. s. - Combining genotype distribution of PPARD T294C and PPARGC1A Gly482Ser in athletes vs. CG n. s. - Performance of 20 m shuttle run test ↑ with genotypes GlyGly, GlySer, SerSer of PPARGC1A polymorphism (*p =* 0.003)
Kikuchi et al. (2017)	MCT1	Detection of the polymorphism of MCT1^1^ in wrestlers vs. CG + its influence on power output and lactate concentration before, during, and after two anaerobic tests	- AA genotype ↑ in wrestlers vs. CG [*p =* 0.037; OR = 1.40; 95% CI (1.02–1.93)] - AA genotype ↓ blood lactate concentrations during (*p =* 0.028), immediately after (*p =* 0.021) and after 10 min regeneration (*p =* 0.048) in anaerobic performance tests vs. TA+TT genotype
Li et al. (2017)	ACTN3	Analysis of ACTN3^1^ genotypes in swimmers vs. CG	- Swimmers vs. CG in allele distribution (R, X) n. s. - RR and RX+XX genotype differs significantly in swimmers vs. CG: → RR and RX+XX: *p < * 0.05 → RX, XX and RR+RX: p > 0.05
Peplonska et al. (2017)	ACE ACTN3 AGT NRF-2 PPARG PPARGC1A TFAM	Detection of the gene variants/ Genotypes of ACE^1^, ACTN3^1^, AGT^1^, NRF-2^1^, PPARG^1^, PPARGC1A^1^ and TFAM^1^in ST and ET vs. CG	- ACE genotypes, NRF-2 rs12594956, TFAM rs2306604 differs significantly in athletes vs. CG: → ACE, D allele: *p =* 0.0095; OR = 1.28; 95% CI (1.06–1.55) → ACE, DD + ID vs. II: *p =* 0.016; OR = 1.48; 95% CI (1.08–2.04) → NRF-2, A allele: *p =* 0.011; OR = 1.28; 95% CI (1.06–1.54) → NRF-2, AA + AC vs. CC: *p =* 0.011; OR = 1.48; 95% CI (1.10–2.00) → TFAM rs2306604, G-allele: *p =* 0.049; OR = 0.83; 95% CI (0.68–1.00) → TFAM rs2306604, GG vs. AG + AA: *p =* 0.69; OR = 0.49; 95% CI (0.49–0.98) - ACTN3 R557X (rs1815739), AGT M235T (rs699), PPARGC1A G482S (rs8192678), PPARG P12A (rs1801282), TFAM rs1973 in athletes vs. CG n. s.
Voisin et al. (2016)	ACVR1B	Analysis of the polymorphism of ACVR1B^1^ in ET, ST, and MT vs. CG	- Genotype distribution in Brazilian and Caucasian (Italian, Polish, Russian) ET vs. CG n. s. - A allele ↑ in Caucasian ST vs. CG (*p =* 0.048) - A allele ↑ in all Caucasian athletes (ST, ET) vs. CG (*p =* 0.024) - Allele and genotype distribution in all Brazilian athletes (ET, ST) vs. CG n. s.
Yang et al. (2017)	ACTN3	Detection of the genotypes of ACTN3^1^ in ST and ET vs. CG + its influence on the athletes' jumping performance in two jumping tests	- Allele distribution differs significantly ST vs. ET and CG (*p =* 0.001, *p < * 0.001) - Genotype distribution differs significantly ST vs. ET and CG in favor of RR genotype: → ST vs. ET (RR vs. XX): *p < * 0.001; OR = 9.7; 95% CI (2.4–39.2) → ST vs. ET (RR + RX vs. XX): *p < * 0.001; OR = 8.7; 95% CI (2.3–32.7) → ST vs. CG (RR vs. XX): *p < * 0.001; OR = 12.6; 95% CI (3.2–50.8) → ST vs. CG (RR + RX vs. XX): *p < * 0.001; OR = 9.62; 95% CI (2.6–35.3) - Significantly better performance in SLJ and SHJ of RR genotype vs. other genotypes in ST (M, F) (*p < * 0.05) - Significantly better performance in SWS (*p < * 0.05), not in SHS of RR genotype vs. other genotypes ET (M) (*p >* 0.05)
Zarebska et al. (2017) (original study)	GSTP1	Detection of the gene variant of GSTP1^1^ in three groups of Russian athletes (ET, MT, ST) vs. CG	- G allele ↑ in all athletes (combined) vs. CG (*p < * 0.0001) - G allele ↑ only in ST vs. CG (*p < * 0.0001) following classification of athletes into three groups (ET, ST, MT) - Genotype distribution for AA, AG, and GG significant different in all athletes (combined) vs. CG (*p < * 0.0001) - Following classification of athletes into three groups (ET, ST, MT): → AA, AG and GG ↑ in ST vs. CG (*p < * 0.0001) → AA, AG and GG ↑ in ET vs. CG (*p =* 0.036) → GG ↑ in ST vs. CG [*p < * 0.0001; OR = 3.92, 95% CI (2.31–7.20)] → GG ↑ in ET vs. CG [*p =* 0.0022, OR = 2.72, 95% CI (1.39–5.29)] - G allele and genotype distribution in MT vs. CG n. s.
Zarebska et al. (2017) (replication study)	GSTP1	Detection of gene variants of GSTP1^1^ in three groups of Polish athletes (ET, MT, ST) vs. CG	- G allele ↑ in all athletes (combined) vs. CG (*p =* 0.010) - G allele ↑ only in ET vs. CG (*p =* 0.009) following classification of athletes into three groups (ET, ST, MT) - Genotype distribution for AA, AG and GG differs significantly in all athletes (combined) vs. CG (*p =* 0.036) - AA, AG, and GG ↑ only in ET vs. CG (*p =* 0.022) - G allele and genotype distribution in MT or ST vs. CG n. s.
Zarebska et al. (2017) (both)	GSTP1	Results of both studies (combined)	- G allele ↑ in all athletes vs. CG (*p < * 0.0001) - G allele ↑ and significant differences in genotype distribution in ET and ST vs. CG after categorization of athletes into three groups (ET, ST, MT) (*p < * 0.0001) - G allele and genotype distribution in MT vs. CG n. s

*↑, significantly upregulated; ↓, significantly downregulated; n. s., not significant (p > 0.05); p, p-value; OR, Odds ratio; CI, confidence interval; r, effect strength*;

1 *see polymorphism/genotypes Table 1, ET, endurance-trained athletes; ST, strength-trained athletes; MT, mixed-trained athletes; GS, Game sports athletes; CG, control group; B, bouldering group; CC, classical climbing group; LDR, long-distance runners; MDR, middle-distance runners; N/A, not specified; SLJ, standing long jump; SHJ, standing high jump; M, Male; F, Female; min, minute*.

**Table 7:** Results of included studies investigating an association between genetic variants and vulnerability to injury.

**Author(s)/year**	**Gene(s)**	**Topic**	**Results [with specification p =, OR =, 95% CI (…), r =]**
Lulińska-Kuklik et al. (2019b)	IL1B IL6 IL6R	Detection of polymorphisms of IL1B^2^, IL6^2^ and IL6R^2^ in IG with diagnosed ACL rupture vs. CG	- IL6 rs1800795 ↑ in IG vs. CG for codominant, recessive, over-dominant, not dominant model → Codominant (GG vs. CG vs. CC): *p =* 0.018; OR = 0.63; 95% CI (0.40–0.99) → Recessive (GG + CG vs. CC): *p =* 0.032; OR = 1.74; 95% CI (1.08–2.81) → Predominant (GG + CC vs. CG): *p =* 0.007; OR = 0.57; 95% CI (0.38–0.83) → Dominant (GG vs. CG + CC): *p >* 0.05 - IL1B rs16944, IL1B rs1143627, IL6R rs2228145 in IG vs. CG n. s.
Lulińska-Kuklik et al. (2019c)	MMP3 MMP8 TIMP2	Detection of polymorphisms of MMP3^2^, MMP8^2^ and TIMP2^2^ in IG with diagnosed ACL rupture vs. CG	- C allele of MMP3 rs591058 and the G allele of MMP3 rs679620 ↑ in IG vs. CG [*p =* 0.021; OR = 1.38; 95% CI (1.05–1.81)] - MMP8 rs11225395, TIMP2 rs5789932 in IG vs. CG n. s.
Lulińska-Kuklik et al. (2019a)	TNC	Detection of polymorphisms of TNC^2^ in IG with diagnosed ACL rupture vs. CG	- TNC rs1330363, TNC rs2104772, TNC rs13321 in IG vs. CG n. s. - Haploid genotypes in IG vs. CG n. s.
Lulińska-Kuklik et al. (2018)	COL5A1	Detection of polymorphisms of COL5A1^2^ in IG with diagnosed ACL rupture vs. CG	- COL5A1 rs13946 ↑ in IG vs. CG for dominant model → Dominant (CC + CT vs. TT): *p =* 0.039, OR = N/A, 95% CI (N/A) - COL5A1 rs12722 in IG vs. CG n. s. - Haploid genotype COL5A1 rs12722-rs13946 in IG vs. CG n. s.
Salles et al. (2018)	FOXP3 FCRL3	Detection of polymorphisms of FOXP3^2^ and FCRL3^2^ in IG with diagnosed tendinopathy vs. CG	- FCRL3 −169 T>C rs7528684 ↑ in IG vs. CG [*p =* 0.04; OR = 2.02; 95% CI (0.10–4.09)] - FOXP3–2383 C>T rs3761549 in IG vs. CG n. s.
Salles et al. (2015)	BMP4 FGF3 FGF10 FGFR1	Detection of polymorphisms of BMP4^2^, FGF3^2^, FGF10^2^ and FGFR1^2^ in IG with diagnosed tendinopathy vs. CG	- GG, GT, and TT genotypes of BMP rs2761884 ↑ in IG vs. CG [*p =* 0.03; OR = N/A, 95% CI (N/A)] - GT + TT genotypes of BMP rs2761884 ↑ in IG vs. CG [*p =* 0.01; OR = 2.39; 95% CI (1.10–5.19)] - T allele of BMP rs2761884 ↑ in IG vs. CG [*p =* 0.007; OR = 2.39; 95% CI (1.16–3.48)] - BMP4 rs2071047, BMP4 rs17563, BMP4 rs2855529, BMP4 rs762642, FGF3 rs7932320, FGF3 rs1893047, FGF3 rs12574452, FGF3 rs4631909, FGF3 rs4980700, FGF10 rs1448037, FGF10 rs900379, FGF10 rs1011814, FGF10 rs593307 and FGFR1 rs13317 in IG vs. CG n. s. - Haploid genotype TTGGA of BMP4 rs2761884-rs2071047-rs17563-rs2855529-rs762642 ↑ in IG vs. CG [*p =* 0.01; OR = 1.92; 95% CI (0.36–10.15)]

*↑, significantly upregulated; n. s., not significant (p > 0.05); p, p-value; OR, Odds ratio; CI, confidence interval; r, effect strength*;

2 *see polymorphisms Table 2, ACL rupture, anterior cruciate ligament rupture; IG, injury group; CG, control group; N/A, not specified*.

### Endurance Performance and Muscle Strength

A total of 13 of the 19 included studies dealt with the relationship between polymorphisms and muscle strength or endurance performance. The majority of the 13 studies investigating endurance performance and muscle strength recruited a group of competitive athletes and subdivided them into specific subgroups, mostly typical in categories with disciplines that are more demanding on endurance or strength capabilities. If athletes could not be clearly assigned to one of these two groups, e.g., because their discipline uses both aerobic and anaerobic energy supply, they were usually assigned to a mixed group of athletes and/or assigned to team sports. In contrast, four of the 13 studies only addressed specific groups of athletes or disciplines, including soccer, climbing, wrestling, and swimming. The results of the four studies that examined only individual disciplines or sports are as follows: Falahati and Arazi could not link any of the genotypes of the ACE variant to the cardiovascular fitness of Iranian footballers (Falahati and Arazi, 2019). Ginszt et al. (2018) and Li et al. (2017) investigated the ACTN3 R577X polymorphism in climbing, bouldering, and swimming and both found an overrepresentation of the RR genotype of the gene variant in their groups of athletes. Kikuchi et al. (2017) also found the MCT1 T1470A rs1049434 polymorphism significantly higher in Japanese elite wrestlers and correlated it with their performance in two anaerobic tests. The remaining nine studies divided their case groups into several sport-specific subgroups and compared them either with a control group and/or among themselves. Ben-Zaken et al. (2015) could not demonstrate a significant association of the IGF-1R 275124 A>C rs1464430 polymorphism with the athlete status of strength and endurance athletes compared to the control group. The athlete status is characterized by the present state of performance regarding endurance exercise capacity and muscle strength in relation to others. However, when comparing the different athlete groups, the AA genotype was significantly overrepresented in endurance and the C allele in strength athletes. The FTO T>A rs9939609 polymorphism and its A allele and AA genotype, respectively, could be clearly and significantly detected in Russian strength athletes and “heavy” athletes of the mixed group (Guilherme et al., 2019). In contrast, exactly this gene variant showed a negative association with the long-distance runners from the endurance group. In 2017, Guilherme and Lancha investigated eight polymorphisms of the CNDP1 and CNDP2 genes and could only assign the two gene variants CNDP1 rs2887 and CNDP2 rs3764509 to muscle strength capacity but could not detect any of the eight variants being significantly altered in endurance athletes (Guilherme and Lancha, 2017). Two groups of authors focused on genes of the PPAR group and their polymorphisms. Peplonska et al. (2017) and Jin et al. (2016) could not relate either PPARD T294C rs2016520 or PPARG P12A rs1801282 polymorphism to elite athlete status. Only the genotypes of PPARGC1A Gly482Ser rs8192678 polymorphism showed a slight significant association with the verified endurance performance of athletes (Jin et al., 2016). Peplonska et al. (2017) tested variants of the genes ACE, ACTN3, NRF-2, AGT, and TFAM in addition to the polymorphisms of the PPAR group (Peplonska et al., 2017). Compared to a control group, the D allele and the DD genotype of the ACE polymorphism and A allele and the AA genotype of NRF-2 rs12594956 were significantly overrepresented in the athlete group. In contrast, the G allele and GG genotype of the TFAM rs2306604 polymorphism showed a negative association with the athletes. In the subsequent comparison of strength and endurance athletes, there was no chance of significantly assigning one or more polymorphisms to a specific subgroup. Voisin et al. (2016) and Yang et al. (2017) identified the A allele of ACVR1B rs2854464 polymorphism in Caucasian sprint and strength athletes (Voisin et al., 2016) and related the RR genotype of ACTN3 R577X polymorphism to Chinese sprint and strength athletes and their jumping performance (Yang et al., 2017). Most recently, the GSTP1 polymorphism was significantly associated with the athlete status of Polish and Russian athletes in an original and a replication study (Zarebska et al., 2017). All results are presented in detail in Table 6.

### Susceptibility to Injury

Six of the 19 included studies investigated injury susceptibility. Five studies reported an association of the studied polymorphisms with soft tissue injuries. Of 30 gene variants analyzed, four polymorphisms were associated with rupture of the anterior cruciate ligament and two with tendinopathy. Significant differences between the case and control groups were found in the distribution of the polymorphisms IL6 rs1800795, MMP3 rs591058 (C allele), and rs679620 (G allele) as well as COL5A1 rs13946, whereby a clearly higher frequency of COL5A1 rs13946 in the case group was only visible in the dominant mode of inheritance (Lulińska-Kuklik et al., 2018, 2019b,c). Furthermore, according to Lulińska-Kuklik et al. the IL6 rs1800795 polymorphism could be associated with cruciate ligament rupture, but the individual inheritance mechanisms revealed inconsistent results (Lulińska-Kuklik et al., 2019b). Furthermore, Lulińska-Kuklik et al. could not provide significant evidence for the gene variants IL1B rs16944 and rs1143627, IL6R rs2228145, MMP8 rs11225395, TIMP2 rs5789932, and COL5A1 rs12722 (Lulińska-Kuklik et al., 2018, 2019b,c). Also, the polymorphisms TNC rs1330363, rs2104772, and rs13321 did not show significant differences between the two groups (Lulińska-Kuklik et al., 2019a). The above-mentioned variants were all investigated in relation to anterior cruciate ligament rupture. For competitive athletes with tendinopathy, the FCRL3 −169 T>C rs7528684 and the BMP4 rs2761884 polymorphism could be used. Their incidence was significantly overrepresented in the case group (Salles et al., 2015, 2018). The authors also reported on the haploid genotype of BMP4 and an association of this with non-inflammatory tendon disease (Salles et al., 2015). No evidence for a genetic influence was found for the variants FOXP3–2383 C>T rs3761549, BMP4 rs2071047, rs17563, rs2855529, rs762642, FGF3 rs7932320, rs1893047, rs12574452, rs4631909, rs4980700, FGF10 rs1448037, rs900379, rs1011814, rs593307, and FGFR1 rs13317 (Salles et al., 2015, 2018). Table 7 summarizes the results of the injury susceptibility studies.

## Discussion

The aim of this systematic review was to assess the effects of genetic variations and polymorphisms on endurance performance, muscle strength and injury susceptibility in competitive sports. The knowledge of performance-related genetic variations could be helpful in optimizing the training content individually, positively influencing athletic performance and compensating for unfavorable genetic predisposition. Results revealed that the IGF-1R 275124 A>C rs1464430 polymorphism was overrepresented in endurance trained athletes and genotypes of PPARGC1A polymorphism correlated with the athletes‘ performance in endurance exercise capacity tests. Moreover, the RR genotype of ACTN3 R577X polymorphism, the C allele of IGF-1R polymorphism and the gene variant FTO T>A rs9939609 and/or their AA-genotype, MCT1 (T1470A rs1049434) and ACVR1B (rs2854464) were linked to muscle strength. Among others, the gene variants of the MMP group (rs591058 and rs679620) as well as the polymorphism COL5A1 rs13946 were associated with susceptibility to injuries of competitive athletes.

Several studies have been conducted investigating the influence of polymorphisms on the endurance performance and muscle strength in competitive athletes. In general, the genetic contribution of the different gene variants of the ACE gene is well-documented. Accordingly, the type II genotype of ACE has been linked with increased endurance performance in previous studies (Hagberg et al., 1998). The DD genotype, on the other hand, was shown to be associated with weight training (Wang et al., 2008). However, this work revealed an equivocal and inconsistent study situation regarding the variants of the ACE gene. For example, Falahati and Arazi could not find any association with cardiovascular determinants of competitive footballers, neither with the II nor with the ID or DD genotype of the ACE gene (Falahati and Arazi, 2019). Likewise, Peplonska et al. found no difference between the subgroups (endurance or strength athletes) in comparison with a sedentary control group (Peplonska et al., 2017). Though, if the intervention groups were combined, the distribution of the D allele and the DD genotype differed significantly from the controls. Thus, in contrast to the previous literature, the polymorphism of ACE could not be associated with endurance performance or muscle strength, but rather with athlete status. In addition, the ACTN3 R577X polymorphism was also frequently addressed by numerous studies. In accordance with previous research on the RR genotype of the ACTN3 polymorphism, Ginszt et al. (2018) were able to significantly associate the R allele and the RR genotypes of the ATCN3 polymorphism with the climbing discipline bouldering that belongs to strength disciplines (North et al., 1999; Yang et al., 2003). In addition, the results of Yang et al. (2003) were supported by an underrepresentation of the X allele and the XX genotype within the boulder group (Ginszt et al., 2018). Further, evidence for a relation of the RR genotype of the ATCN3 R577X polymorphism to the muscle strength of competitive athletes is provided by Peplonska et al. (2017). In this case, this genotype was detected significantly more frequently in sprint and strength athletes and could be associated with their jumping performance. Li et al. referred to both strength and endurance-related phenotypes in swimmers (Li et al., 2017). The results of their study showed that both the RR genotype and the RX+XX genotype of the ACTN3 polymorphism are significantly overrepresented in these athletes compared to a control group. However, a classification of the RX genotype is difficult, since scientific evidence is rare. Further research is needed to classify the effects of this genotype more precisely (Guth and Roth, 2013). In contrast, the study of Peplonska et al. found no significant differences regarding the ACTN3 R577X polymorphism in athletes compared to control subjects (Peplonska et al., 2017). There is already some evidence regarding an association of PPAR polymorphism with increased endurance exercise capacity. For example, Maciejewska et al. demonstrated that the G allele is associated with elite endurance status as compared with controls (Maciejewska et al., 2012). However, neither Jin et al. (2016) nor Peplonska et al. (2017) could validate this relationship in their studies. Thus, the PPARGC1A Gly482Ser polymorphism was only associated with endurance performance in one of seven performance tests (Jin et al., 2016). In both studies, this could mainly be attributed to an ethnic and sports discipline-related heterogeneity and small sample sizes of the studied athlete groups, since the selection of a homogeneous group could be of great importance for the detection of genetic polymorphisms. Peplonska et al. (2017) also focused on NRF polymorphisms, which are believed to be related to improved endurance exercise capacity. They found a significant association of the A allele and the AA genotype of NRF-2 rs12594956 in the group of athletes. Furthermore, they examined the frequencies of variants of the AGT and TFAM genes. The distribution of the G allele and GG genotype of TFAM rs2306604 differed significantly between the athlete and control groups (Peplonska et al., 2017). In this case, the OR must be considered more closely: Although the difference between athletes and controls was significant in the distribution of TFAM rs2306604, the TFAM polymorphism showed a negative correlation with athlete status (OR < 1) (Peplonska et al., 2017). Thus, the deficiency of TFAM rs2306604 seems to increase the chance of being an elite athlete. For the AGT M235T rs699 polymorphism, which was also investigated, no significant differences between the groups could be found (Peplonska et al., 2017). A similar phenomenon was demonstrated regarding the polymorphism of the FTO gene. Gene variants of the FTO gene have been associated with increased obesity in the past (Peng et al., 2011). In the study by Guilherme et al. (2019), the AA genotype of the FTO T>A rs9939609 polymorphism shows, on the one hand, a positive association with Russian power and strength athletes (OR > 1) and, on the other hand, a negative association with long-distance runners (OR < 1). Therefore, it can be assumed that the investigated polymorphism of the FTO gene might have a disadvantageous effect on the endurance performance of athletes. Furthermore, the distribution of the receptor of IGF-1 (IGF-1R) or its polymorphism was investigated in strength and endurance athletes compared to a control group (Ben-Zaken et al., 2015). The results of Ben-Zaken et al. (2015) showed a significant difference in the prevalence of the gene variant of IGF-1R between endurance and strength athletes, but not in comparison to controls. The AA genotype of IGR-1R 275124 A>C rs1464430 polymorphism was assigned to endurance athletes and the C allele to strength athletes. For muscle strength, other genes and their polymorphisms were discussed as key genes. Both the G allele of CNDP2 rs3764509 and the A allele of CNDP1 rs2887 were two of eight investigated variants found in connection with strength athletes (Guilherme and Lancha, 2017). In terms of the CNDP1 rs2887 polymorphism, however, contradictory results were presented. On the one hand, Guilherme and Lancha (2017) report that the A allele was significantly overrepresented in strength athletes compared to the mixed group of athletes. On the other hand, however, a look at the OR suggests that there was a slight correlation between the CNDP1 rs2887 polymorphism and the strength athletes (OR < 1) and thus appears rather unfavorable for this group. Moreover, Kikuchi et al. (2017) found the polymorphism MCT1 T1470A rs1049434 or its AA genotype significantly higher in wrestlers as opposed to a control group. These results were confirmed by low lactate levels of the elite wrestlers in two performance tests. The authors suggest that the MCT1 AA genotype improves blood lactate transport during anaerobic exercise and/or recovery and is associated with successful high-intensity, strength-oriented, intermittent athletic performance (Kikuchi et al., 2017). Voisin et al. (2016) also found the A allele of ACVR1B rs2854464 polymorphism significantly higher in Caucasian strength athletes compared to controls. Most recently, two studies by Voisin et al. (2016) did not provide clear results. The GSTP1 c.313 A>G polymorphism and its G allele or GG genotype was found more frequently expressed in both endurance and strength athletes compared to a control group in the original study. Conversely, the replication study detected the mentioned allele or these genotypes only in endurance athletes (Zarebska et al., 2017). In the combined analysis, however, the results of the original study were confirmed. Furthermore, no comparison between the strength and endurance groups was made, so that a clear assignment of the GSTP1 c.313 A>G polymorphism was not possible. Thus, it can only be associated with athlete status (Zarebska et al., 2017). Well-known polymorphisms could be confirmed for the injury susceptibility of competitive athletes. Lulińska-Kuklik et al. examined not only the gene variants of the genes of MMP3 (Lulińska-Kuklik et al., 2019c), MMP8 (Lulińska-Kuklik et al., 2019c), TNC (Lulińska-Kuklik et al., 2019a), and COL5A1 (Lulińska-Kuklik et al., 2018) but also a polymorphism of the TIMP2 (Lulińska-Kuklik et al., 2019c) gene. Furthermore, they also analyzed polymorphisms of IL1B, IL6, and IL6R in connection with anterior cruciate ligament rupture (Lulińska-Kuklik et al., 2019b). For the variants MMP3 rs591058 and rs679620 an association with the susceptibility to injury of athletes was validated in accordance with prior studies (Raleigh et al., 2009). COL5A1 rs13946 was only associated with anterior cruciate ligament rupture in the dominant mode of inheritance. However, other studies do not clearly confirm this relation (Mokone et al., 2006; Posthumus et al., 2009; September et al., 2009). Moreover, the results of the investigated variants of the TNC gene could not confirm the existing literature since none of the injured competitive athletes showed a higher frequency of the three investigated polymorphisms of the TNC gene compared to the controls (Lulińska-Kuklik et al., 2019b). Similarly, for the variant TIMP2 rs4789932 no significant differences were found between the case and control group (Lulińska-Kuklik et al., 2019c). Interestingly, regarding interleukins the presence of IL6 rs1800795 in the co- and overdominant inheritance mode reduced the risk of anterior cruciate ligament rupture (OR < 1). The IL6 rs1800795 polymorphism can therefore act as a protective factor under these circumstances. In the recessive model, however, IL6 rs1800795 showed an increased probability for the occurrence of anterior cruciate ligament rupture (OR > 1) (Lulińska-Kuklik et al., 2019b). Thus, a general directional statement for the injury preventive effects of the IL6 gene and its polymorphisms cannot be formulated due to divergent results depending on the mode of inheritance. Particularly, the IL6 rs1800795 polymorphism points to the complexity of inheritance processes and highlights the risk of jumping to conclusions with respect to generally valid statements about the influence of a gene and/or its polymorphisms. No significant results were obtained for the variants of the IL1B and IL6R genes (Lulińska-Kuklik et al., 2019b). Further, Salles et al. revealed an association between the inflammatory response of the immune system and the susceptibility to injury (Salles et al., 2015, 2018). A significant effect of FCRL3 −169 T>C rs7528684 polymorphism was demonstrated for athletes with inflammatory tendinopathy, but a significant distribution for the variant of FOXP3 could not be provided (Salles et al., 2018). Regarding non-inflammatory tendon disease, a genetic contribution of the variants of the genes BMP4, FGF3, FGF10, and FGFR1 was additionally investigated. The BMP4 rs2761884 polymorphism was identified as a risk factor for the development of tendinopathy. For the polymorphisms of the genes around FGF3, FGF10, and FGFR1 no significant results could be found (Salles et al., 2015). In addition to the analysis of individual polymorphisms, some authors also investigated haploid genotypes or combinations of analyzed polymorphisms in connection with the susceptibility of athletes to injury. Lulińska-Kuklik et al. (2019a) analyzed the haploid genotypes of the TNC polymorphisms and different combinations of interleukin variants but failed to provide significant results in any analysis. However, Salles et al. (2018), showed a significant gene-gene interaction of the variants of the genes of FCRL3 and FOXP3 as a risk factor for tendinopathy. On top of that, the linkage of the five polymorphisms of the BMP4 gene investigated showed a significant association with tendinopathy in the TTGGA genotype (Salles et al., 2015). Further, the haploid genotype of COL5A1 rs12722-rs13946 was significantly overrepresented in the dominant model in controls as opposed to the athlete group. Thus, this haplotype could be interpreted as a protective factor (Lulińska-Kuklik et al., 2018). In summary, the analysis of haplotypes or the interaction of genes and their variants has not received sufficient attention in the current literature. However, the need for the analysis of haplotypes or the interaction of gene variants is based on the biological interaction processes for the development of athletic performance.

Finally, we want to point out some limitations and discuss the quality of the included studies. This review aims to provide an overall approach and therefore focuses on the parent categories endurance, muscle strength and injury susceptibility. Future studies should identify and differentiate the effects of genetic predisposition on more specified performance-related factors such as aerobic capacity or explosive strength. The search strategy of the current work was limited to the two databases PubMed and Web of Science and not extended to other databases. Therefore, possibly not all existing literature on genetic polymorphisms with an influence on performance and susceptibility to injury in competitive sports has been compiled. Although, many of the included studies were able to map associations of their genetic polymorphisms in relation to athletes' performance and vulnerability to injury, the quality of the studies must be considered to evaluate their validity. Using the RoBANS instrument (Kim et al., 2013), all bias possibilities of the included cross-sectional and case-control studies were assessed. A large proportion of the studies clearly defined exposure and chose appropriate measurement methods (Ben-Zaken et al., 2015; Salles et al., 2015; Voisin et al., 2016; Peplonska et al., 2017; Lulińska-Kuklik et al., 2018, 2019a,b,c; Guilherme et al., 2019). The reporting also included both significant and non-significant results as far as possible and the full presentation of all results. Only a few studies could not be clearly sorted into these categories (Voisin et al., 2016; Kikuchi et al., 2017) (for details see Table 5). Considerable shortcomings were found in the category “blinding of the result evaluation.” Only three studies referred to a blinding of the examiners (Yang et al., 2017; Lulińska-Kuklik et al., 2018; Falahati and Arazi, 2019), while for the remaining 16 studies a high potential of bias was found. Likewise, the choice of study participants and the handling of confounders were not adequate in most of the studies, so the results of the affected studies must be treated with caution due to insufficient and inadequate definition of the control groups as well as providing a lack of information on control subjects, including age and gender distribution (Ben-Zaken et al., 2015; Jin et al., 2016; Voisin et al., 2016; Yang et al., 2017). Furthermore, the transferability of the results was not always guaranteed. For example, Falahati and Arazi (2019), Kikuchi et al. (2017) as well as Lulińska-Kuklik et al. (2018) and Salles et al. (2015) limited their study population to male athletes, which, on the one hand, excluded gender as a possible disruptive factor, but, on the other hand, clearly limited the representativeness and thus the transferability of their study results to the target population of competitive athletes. Finally, only a total of nine studies showed suitable design methods or statistical procedures to counter disturbing factors such as age and gender (Ben-Zaken et al., 2015; Jin et al., 2016; Salles et al., 2018). For the reasons mentioned above, more than half of the studies had to be rated with a high bias potential in the categories “selection of participants” and “disturbing factors.” Therefore, the quality assessment of the included studies points to a potential limitation of the result evaluation due to the unclear or high bias potential of many studies in some categories. In addition, comparability of the studies is limited due to the different design in the structure, analysis, and evaluation. Some authors restricted their study population to a single type of exercise (Kikuchi et al., 2017; Li et al., 2017; Ginszt et al., 2018; Falahati and Arazi, 2019) while others formed heterogeneous cohorts of athletes from several disciplines (Guilherme and Lancha, 2017; Yang et al., 2017; Zarebska et al., 2017; Guilherme et al., 2019). Since the characteristics of endurance performance, muscle strength and injury susceptibility vary in children, adolescents, and seniors, these groups were excluded to increase the generalizability of the results by focusing on a normal adult age. Further, we have chosen the inclusion of athletes broadly because in some sports the age for top sporting performance already is at 18 years, in others, it is at an older middle age. The genetic prerequisites should be reflected in all these age groups. However, the wide age range may have influenced the results. Furthermore, the comparability of the study results is limited by the fact that several studies examine the same gene but different polymorphisms (Salles et al., 2015; Guilherme and Lancha, 2017; Lulińska-Kuklik et al., 2019a). In addition, an association of a gene with exercise performance or injury susceptibility should preferably be assessed in connection with the respective polymorphism. Consequently, in future studies comparability of results should be assessed on the level of gene variants and not only on the level of genes. Furthermore, it was sometimes difficult to assign the examined individual sports disciplines to either the category of endurance performance or the category of muscle strength. For future investigations, it would be useful to create clear, uniform and superordinate definitions for subgroups according to metabolic and energetic requirements. This also seems promising and purposeful regarding polymorphisms and their specific influence on metabolic pathways and regeneration processes. Since the occurrence of genetic polymorphisms depends on ethnicity, the origin of the study population should always be considered in future studies. For example, Voisin et al. (2016) investigated the distribution of the ACVR1B rs2854464 variant in a Caucasian and a Brazilian athlete group compared to respective control groups of the same origin. ACVR1B rs2854464 polymorphism was significantly overrepresented in Caucasian strength and sprint athletes and significantly underrepresented in Brazilian strength and sprint athletes. In addition, the performance level of the athletes also plays a major role—even in competitive sports. Some of the included studies additionally subdivided the competitive athletes according to their competition level (national vs. international) and compared the distribution of the respective polymorphism between the subgroups or with a control group. For example, Guilherme and Lancha (2017) found no significant difference in the distribution of CNDP2 rs6566810 between endurance athletes and the control group, but this polymorphism was significantly overrepresented in international endurance athletes compared to controls after subdivision into subgroups. These results indicate that polymorphisms are not only distributed differently within sport groups but can also differ in their frequency among performance classes. Finally, we want to point out some current developments. A recent study showed that in addition to the previously described association with muscle strength, the allele distribution of ACTN3's R577X polymorphism also varied significantly depending on the field position in professional football players (Clos et al., 2021). Thus, analyzing genetic characteristics of football players may be useful when evaluating performance capability and optimizing training protocols (…). In contrast to the COL5A1 rs13946 identified in this review, a current study reveals that COLIA1 + 1245 G > T Sp1 binding site does not seem to be linked to the risk for soft tissue injuries (Shukla et al., 2020). This indicates that the effects of different collagen types on the susceptibility to soft tissue injuries may differ strongly. Moreover, a novel study indicates that the calculation of the total genetic score may be used as an instrument to enhance the performance in top athletes (Amato et al., 2018).

In summary, the results revealed that the IGF-1R 275124 A>C rs1464430 polymorphism was overrepresented in endurance-trained athletes. Further, genotypes of PPARGC1A polymorphism correlated with performance in endurance exercise capacity tests in athletes. For muscle strength, the current systematic review process could confirm a well-known and already well-studied polymorphism: The RR genotype of ACTN3 R577X polymorphism showed a positive association with strength athletes in several studies. Moreover, studies provide evidence for the overrepresentation of the C allele of the polymorphism of IGF-1R and the gene variant FTO T>A rs9939609 and/or their AA genotype in strength athletes. In addition, the newly sprouting gene variants of MCT1 (T1470A rs1049434) and ACVR1B (rs2854464) were also positively associated with strength performance. Among others, the gene variants of the MMP group (rs591058 and rs679620) and the polymorphism COL5A1 rs13946 were associated with susceptibility to injuries of competitive athletes. In accordance with previous research, the gene variants of the MMP group (rs591058 and rs679620) and the polymorphism COL5A1 rs13946 could be linked to the injury susceptibility of athletes in the dominant mode of inheritance. The association of the TNC polymorphism with injury susceptibility could not be supported by recent studies. Few studies have been available on the FOXP3 and FCLR3 polymorphism, BMP4 and the FGF group. In this case, both the FCLR3 −169 T>C rs7528684 and the BMP4 rs2761884 gene variant were found more frequently in connection with injury susceptibility in competitive athletes. Finally, depending on the mode of inheritance the polymorphism IL6 rs1800795 could also be associated with the susceptibility to injuries.

## Conclusions

In conclusion, specific genetic variants and polymorphisms were identified that are associated with exercise performance as well as injury susceptibility. With the knowledge about the existence of specific polymorphisms, which can be risk factors for injuries, the healing process can be positively influenced, the endurance or strength training can be planned specifically, and the athlete can be optimally supported with the right amount of training. Not only in individual disciplines, but also in team sports, the knowledge of an individual genetic profile is useful to derive an optimized one-to-one training. For athletes who have an increased likelihood of musculoskeletal injuries due to an unfavorable genetic predisposition, specific individualized injury prevention programs should be created, and weaknesses should be compensated preventively through targeted muscular strengthening, mobilization, and physical therapy. However, recent research reveals that genetic testing is currently still unsuitable as a tool for talent identification due to problems in the precise differentiation between elite athletes and nonathletic controls (Pickering and Kiely, 2020). This systematic review shows that there is an ongoing need for high-quality future studies for endurance, muscle strength and susceptibility to injuries to investigate possible polymorphisms that can play a decisive role in competitive sports.

## Data Availability Statement

The original contributions presented in the study are included in the article/Supplementary Material, further inquiries can be directed to the corresponding author/s.

## Author Contributions

KA and MA: conceptualization and methodology. MA, KA, KK, and KZ: validation and writing—review and editing. MA: formal analysis, investigation, data curation, writing—original draft preparation, and visualization. KA and KK: supervision. KA: project administration. All authors have read and agreed to the published version of the manuscript.

## Conflict of Interest

The authors declare that the research was conducted in the absence of any commercial or financial relationships that could be construed as a potential conflict of interest.

## Abbreviations

ACE: angiotensin converting enzyme
ACTN3: alpha-actinin 3
ACVR1B: activin receptor type 1B
AGT: angiotensinogen
BMP: bone morphogenetic protein
CI: confidence interval
CNDP: carnosine dipeptidase
COL1A1: alpha-1 type I collagen
COL5A1: alpha-1 type V collagen
DNA: deoxyribonucleic acid
FCRL3: Fc receptor-like protein 3
FGF: fibroblast growth factor
FGFR1: fibroblast growth factor receptor 1
FOXP3: the forkhead box protein P3
FTO: fat mass and obesity associated protein
GSTP1: glutathione S-transferase P1
HIF-1: hypoxia-inducing factor 1
IGF-1: insulin-like growth factor 1
IGF-1R: insulin-like growth factor 1 receptor
IL1B: interleukin-1B
IL6: interleukin-6
IL6R: interleukin-6 receptor
MCT1: monocarboxylate transporter 1
MMP: matrix metalloprotease
N/A: not listed
NRF-2: nuclear respiratory factor 2
OR: odds ratio
PPAR: peroxisome proliferator activated receptor(s)
PPARG: peroxisome proliferator activated receptor gamma
r: effect strength
ROB: risk-of-bias
ROBANS: risk-of-bias tool for non-randomized studies
TFAM: mitochondrial transcription factor A
TIMP2: tissue inhibitors of metalloproteinase 2
TNC: tenascin C
VEGF: vascular endothelial growth factor.

## References

[B1] Ahmetov I. I. Druzhevskaya A. M. Lyubaeva E. V. Popov D. V. Vinogradova O. L. Williams A. G. (2011). The dependence of preferred competitive racing distance on muscle fibre type composition and ACTN3 genotype in speed skaters. Exp. Physiol. 96, 1302–1310. 10.1113/expphysiol.2011.060293 21930675

[B2] Ahmetov I. I. Egorova E. S. Gabdrakhmanova L. J. Fedotovskaya O. N. (2016). Genes and athletic performance: an update. Med. Sport Sci. 61, 41–54. 10.1159/000445240 27287076

[B3] Akhmetov I. I. Astranenkova I. V. Rogozkin V. A. (2007). Association of PPARD gene polymorphism with human physical performance. Mol. Biol. 41, 852–857. 10.1134/S002689330705010X 18240567

[B4] Amato A. Messina G. Contro V. Sacco A. Prioa P. (2018). Total genetic score: An instrument to improve the performcance in elite athletes. Acta Med. Medit. 6, 1857–1862. 10.19193/0393-6384_2018_6_287.

[B5] Bauer G. (2013). Sportverletzungen. Unfallchirurg. 116, 486–487. 10.1007/s00113-013-2370-9 23744177

[B6] Ben-Zaken S. Meckel Y. Nemet D. Eliakim A. (2015). IGF-I receptor 275124AC (rs1464430) polymorphism and athletic performance. J. Sci. Med. Sport. 18, 323–327. 10.1016/j.jsams.2014.03.007 24745653

[B7] Brent A. E. Tabin C. J. (2004). FGF acts directly on the somitic tendon progenitors through the Ets transcription factors Pea3 and Erm to regulate scleraxis expression. Development 131, 3885–3896. 10.1242/dev.01275 15253939

[B8] Chiquet-Ehrismann R. Tucker R. P. (2011). Tenascins and the importance of adhesion modulation. Cold Spring Harb. Perspect. Biol. 3, 1–19. 10.1101/cshperspect.a004960 PMC3101840 21441591

[B9] Clarkson P. M. Hoffman E. P. Zambraski E. Gordish-Dressman H. Kearns A. Hubal M.. (2005). ACTN3 and MLCK genotype associations with exertional muscle damage. J. Appl. Physiol. 99, 564–569. 10.1152/japplphysiol.00130.2005 15817725

[B10] Clos E. Pruna R. Lundblad M. Artells R. Maffulli N. (2021). ACTN3's R577X single nucleotide polymorphism allele distribution differs significantly in professional football players according to their field position. Med Princ Pract. 30, 92–97. 10.1159/000509089 32492691 PMC7923889

[B11] Collins M. (2009). Genetics and Sports. Basel: Karger. 10.1159/isbn.978-3-8055-9028-0

[B12] Czarnik-Kwaśniak J. Kwaśniak K. Tabarkiewicz J. (2019). How genetic predispositions may have impact on injury and success in sport. Eur J Clin Exp Med. 16, 366–375. 10.15584/ejcem.2018.4.16

[B13] Danser A. H. Schalekamp M. A. Bax W. A. van den Brink A. M. Saxena P. R. Riegger G. A.. (1995). Angiotensin-converting enzyme in the human heart. Effect of the deletion/insertion polymorphism. Circulation 92, 1387–1388. 10.1161/01.CIR.92.6.1387 7664416

[B14] Döring F. Onur S. Fischer A. Boulay M. R. Pérusse L. Rankinen T.. (2010). A common haplotype and the Pro582Ser polymorphism of the hypoxia-inducible factor-1alpha (HIF1A) gene in elite endurance athletes. J. Appl. Physiol. 108, 1497–1500. 10.1152/japplphysiol.01165.2009 20299614

[B15] Dubouchaud H. Butterfield G. E. Wolfel E. E. Bergman B. C. Brooks G. A. (2000). Endurance training, expression, and physiology of LDH, MCT1, and MCT4 in human skeletal muscle. Am. J. Physiol. Endocrinol. Metab. 278, E571–E579. 10.1152/ajpendo.2000.278.4.E571 10751188

[B16] Eynon N. Duarte J. A. Oliveira J. Sagiv M. Yamin C. Meckel Y.. (2009a). ACTN3 R577X polymorphism and Israeli top-level athletes. Int. J. Sports Med. 30, 695–698. 10.1055/s-0029-1220731 19544227

[B17] Eynon N. Hanson E. D. Lucia A. Houweling P. J. Garton F. North K. N.. (2013). Genes for elite power and sprint performance: ACTN3 leads the way. Sports Med. 43, 803–817. 10.1007/s40279-013-0059-4 23681449

[B18] Eynon N. Sagiv M. Meckel Y. Duarte J. A. Alves A. J. Yamin C.. (2009b). NRF2 intron 3 A/G polymorphism is associated with endurance athletes' status. J. Appl. Physiol. 107, 76–79. 10.1152/japplphysiol.00310.2009 19478192

[B19] Falahati A. Arazi H. (2019). Association of ACE gene polymorphism with cardiovascular determinants of trained and untrained Iranian men. Genes Environ. 41:8. 10.1186/s41021-019-0126-7 30988833 PMC6448307

[B20] Ginszt M. Michalak-Wojnowska M. Gawda P. Wojcierowska-Litwin M. Korszeń-Pilecka I. Kusztelak M.. (2018). ACTN3 genotype in professional sport climbers. J. Strength Cond. Res. 32, 1311–1315. 10.1519/JSC.0000000000002457 29401200 PMC5916482

[B21] Giustiniani A. Battaglia G. Messina G. Morello H. Guastella S. Iovane A.. (2021). Transcranial alternating current stimulation (tACS) does not affect sports people's explosive power: a pilot study. Front Hum Neurosci. 15:640609. 10.3389/fnhum.2021.640609 33994980 PMC8116517

[B22] Guilherme J. P. F. Egorova E. S. Semenova E. A. Kostryukova E. S. Kulemin N. A. Borisov O. V.. (2019). The A-allele of the FTO gene rs9939609 polymorphism is associated with decreased proportion of slow oxidative muscle fibres and overrepresented in heavier athletes. J. Strength Cond. Res. 33, 691–700. 10.1519/JSC.0000000000003032 30694969

[B23] Guilherme J. P. L. F. Lancha A. H. (2017). Single nucleotide polymorphisms in carnosinase genes (CNDP1 and CNDP2) are associated with power athletic status. Int. J. Sport Nutr. Exerc. Metab. 27, 533–542. 10.1123/ijsnem.2017-0098 28871847

[B24] Guilherme J. P. L. F. Tritto A. C. C. North K. N. Lancha Junior A. H. Artioli G. G. (2014). Genetics and sport performance: current challenges and directions to the future. Rev. bras. educ. fís. esporte 28, 177–193. 10.1590/S1807-55092014000100177

[B25] Guth L. M. Roth S. M. (2013). Genetic influence on athletic performance. Curr. Opin. Pediatr. 25, 653–658. 10.1097/MOP.0b013e3283659087 24240283 PMC3993978

[B26] Hagberg J. M. Ferrell R. E. McCole S. D. Wilund K. R. Moore G. E. (1998). VO_2_ max is associated with ACE genotype in postmenopausal women. J. Appl. Physiol. 85, 1842–1846. 10.1152/jappl.1998.85.5.1842 9804589

[B27] Harris R. C. Wise J. A. Price K. A. Kim H. J. Kim C. K. Sale C. (2012). Determinants of muscle carnosine content. Amino Acids. 43, 5–12. 10.1007/s00726-012-1233-y 22327512 PMC3374101

[B28] Hayes J. D. Flanagan J. U. Jowsey I. R. (2005). Glutathione transferases. Annu. Rev. Pharmacol. Toxicol. 45, 51–88. 10.1146/annurev.pharmtox.45.120403.095857 15822171

[B29] He Z. Hu Y. Feng L. Lu Y. Liu G. Xi Y.. (2007). NRF2 genotype improves endurance capacity in response to training. Int. J. Sports Med. 28, 717–721. 10.1055/s-2007-964913 17357964

[B30] Jin H. J. Hwang I. W. Kim K. C. Cho H. I. Park T. H. Shin Y. A.. (2016). Is there a relationship between PPARD T294C/PPARGC1A Gly482Ser variations and physical endurance performance in the Korean population? Genes Genom 38, 389–395. 10.1007/s13258-015-0380-4

[B31] Kaynak M. Nijman F. van Meurs J. Reijman M. Meuffels D. E. (2017). Genetic variants and anterior cruciate ligament rupture: a systematic review. Sports Med. 47, 1637–1650. 10.1007/s40279-017-0678-2 28102489 PMC5507974

[B32] Kelly D. P. Scarpulla R. C. (2004). Transcriptional regulatory circuits controlling mitochondrial biogenesis and function. Genes Dev. 18, 357–368. 10.1101/gad.1177604 15004004

[B33] Kikuchi N. Fuku N. Matsumoto R. Matsumoto S. Murakami H. Miyachi M.. (2017). The association between MCT1 T1470A polymorphism and power-oriented athletic performance. Int. J. Sports Med. 38, 76–80. 10.1055/s-0042-117113 27813046

[B34] Kim S. Y. Park J. E. Lee Y. J. Seo H. J. Sheen S. S. Hahn S.. (2013). Testing a tool for assessing the risk of bias for nonrandomized studies showed moderate reliability and promising validity. J. Clin. Epidemiol. 66, 408–414. 10.1016/j.jclinepi.2012.09.016 23337781

[B35] Li Y. C. Wang L. Q. Yi L. Y. Liu J. H. Hu Y. Lu Y. F.. (2017). ACTN3 R577X genotype and performance of elite middle-long distance swimmers in China. Biol. Sport. 34, 39–43. 10.5114/biolsport.2017.63731 28416896 PMC5377559

[B36] Lippi G. Longo U. G. Maffulli N. (2010). Genetics and sports. Br. Med. Bull. 93, 27–47. 10.1093/bmb/ldp007 19208613

[B37] Löffler G. Petrides P. (2013). Biochemie und Pathobiochemie. Springer.

[B38] Lucia A. Gómez-Gallego F. Santiago C. Bandrés F. Earnest C. Rabadán M.. (2006). ACTN3 genotype in professional endurance cyclists. Int. J. Sports Med. 27, 880–884. 10.1055/s-2006-923862 16612741

[B39] Lulińska-Kuklik E. Laguette M. J. N. Moska W. Weber-Rajek M. Ficek K. Puchala R.. (2019a). Are TNC gene variants associated with anterior cruciate ligament rupture susceptibility? J. Sci. Med. Sport. 22, 408–412. 10.1016/j.jsams.2018.10.003 30528246

[B40] Lulińska-Kuklik E. Maculewicz E. Moska W. Ficek K. Kaczmarczyk M. Michałowska-Sawczyn M.. (2019b). Are IL1B, IL6 and IL6R gene variants associated with anterior cruciate ligament rupture susceptibility? J. Sports Sci. Med. 18, 137–145. 30787661 PMC6370956

[B41] Lulińska-Kuklik E. Rahim M. Domańska-Senderowska D. Ficek K. Michałowska-Sawczyn M. Moska W.. (2018). Interactions between COL5A1 gene and risk of the anterior cruciate ligament rupture. J. Hum. Kinet. 62, 65–71. 10.1515/hukin-2017-0177 29922378 PMC6006531

[B42] Lulińska-Kuklik E. Rahim M. Moska W. Maculewicz E. Kaczmarczyk M. Maciejewska-Skrendo A.. (2019c). Are MMP3, MMP8 and TIMP2 gene variants associated with anterior cruciate ligament rupture susceptibility? J. Sci. Med. Sport. 22, 753–757. 10.1016/j.jsams.2019.01.014 30755371

[B43] Macarthur D. G. North K. N. (2005). Genes and human elite athletic performance. Hum. Genet. 116, 331–339. 10.1007/s00439-005-1261-8 15726413

[B44] Maciejewska A. Sawczuk M. Cieszczyk P. Mozhayskaya I. A. Ahmetov I. I. (2012). The PPARGC1A gene Gly482Ser in polish and Russian athletes. J. Sports Sci. 30, 101–113. 10.1080/02640414.2011.623709 22122487

[B45] Maffulli N. Margiotti K. Longo U. G. Loppini M. Fazio V. M. Denaro V. (2013). The genetics of sports injuries and athletic performance. Muscles Ligaments Tendons J. 3, 173–189. 24367777 PMC3838326

[B46] McPhee J. S. Perez-Schindler J. Degens H. Tomlinson D. Hennis P. Baar K.. (2011). HIF1A P582S gene association with endurance training responses in young women. Eur. J. Appl. Physiol. 111, 2339–2347. 10.1007/s00421-011-1869-4 21344271

[B47] Moher D. Liberati A. Tetzlaff J. Altman D. G. (2009). Preferred reporting items for systematic reviews and meta-analyses: the PRISMA statement. PLoS Med. 6:e1000097. 10.1371/journal.pmed.1000097 19621072 PMC2707599

[B48] Mokone G. G. Schwellnus M. P. Noakes T. D. Collins M. (2006). The COL5A1 gene and Achilles tendon pathology. Scand. J. Med. Sci. Sports. 16, 19–26. 10.1111/j.1600-0838.2005.00439.x 16430677

[B49] Montgomery H. E. Marshall R. Hemingway H. Myerson S. Clarkson P. Dollery C.. (1998). Human gene for physical performance. Nature 393, 221–222. 10.1038/30374 9607758

[B50] North K. N. Yang N. Wattanasirichaigoon D. Mills M. Easteal S. Beggs A. H. (1999). A common nonsense mutation results in alpha-actinin-3 deficiency in the general population. Nat. Genet. 21, 353–354. 10.1038/7675 10192379

[B51] Peng S. Zhu Y. Xu F. Ren X. Li X. Lai M. (2011). FTO gene polymorphisms and obesity risk: a meta-analysis. BMC Med. 9:71. 10.1186/1741-7015-9-71 21651756 PMC3118373

[B52] Peplonska B. Adamczyk J. G. Siewierski M. Safranow K. Maruszak A. Sozanski H.. (2017). Genetic variants associated with physical and mental characteristics of the elite athletes in the polish population. Scand. J. Med. Sci. Sports. 27, 788–800. 10.1111/sms.12687 27140937

[B53] Philippou A. Halapas A. Maridaki M. Koutsilieris M. (2007). Type I insulin-like growth factor receptor signaling in skeletal muscle regeneration and hypertrophy. J. Musculoskelet. Neuronal Interact. 7, 208–218. 17947802

[B54] Pickering C. Kiely J. (2020). Can genetic testing predict talent? A case study of 5 elite athletes. Int. J. Sports Physiol. Perform. 16, 429–434. 10.1123/ijspp.2019-0543 33271500

[B55] Pokrywka A. Kaliszewski P. Majorczyk E. Zembroń-Łacny A. (2013). Genes in sport and doping. Biol. Sport. 30, 155–161. 10.5604/20831862.1059606 PMC3944571 24744482

[B56] Posthumus M. Collins M. (2016). Genetics and Sports. Basel: Karger. 10.1159/isbn.978-3-318-03011-2

[B57] Posthumus M. Collins M. Cook J. Handley C. J. Ribbans W. J. Smith R. K. W.. (2010). Components of the transforming growth factor-beta family and the pathogenesis of human Achilles tendon pathology–a genetic association study. Rheumatology 49, 2090–2097. 10.1093/rheumatology/keq072 20360039

[B58] Posthumus M. September A. V. O'Cuinneagain D. van der Merwe W. Schwellnus M. P. Collins M. (2009). The COL5A1 gene is associated with increased risk of anterior cruciate ligament ruptures in female participants. Am. J. Sports Med. 37, 2234–2240. 10.1177/0363546509338266 19654427

[B59] Pruna R. Artells R. Lundblad M. Maffulli N. (2016). Genetic biomarkers in non-contact muscle injuries in elite soccer players. Knee Surg. Sports Traumatol. Arthrosc. 25, 3311–3318. 10.1007/s00167-016-4081-6 27085366

[B60] Raleigh S. M. van der Merwe L. Ribbans W. J. Smith R. K. W. Schwellnus M. P. Collins M. (2009). Variants within the MMP3 gene are associated with achilles tendinopathy: possible interaction with the COL5A1 gene. Br. J. Sports Med. 43, 514–520. 10.1136/bjsm.2008.053892 19042922

[B61] Rigat B. Hubert C. Alhenc-Gelas F. Cambien F. Corvol P. Soubrier F. (1990). An insertion/deletion polymorphism in the angiotensin I-converting enzyme gene accounting for half the variance of serum enzyme levels. J. Clin. Invest. 86, 1343–1346. 10.1172/JCI114844 1976655 PMC296868

[B62] Salles J. I. Amaral M. V. Aguiar D. P. Lira D. A. Quinelato V. Bonato L. L.. (2015). BMP4 and FGF3 haplotypes increase the risk of tendinopathy in volleyball athletes. J. Sci. Med. Sport. 18, 150–155. 10.1016/j.jsams.2014.02.011 24661680

[B63] Salles J. I. Lopes L. R. Duarte M. E. L. Morrissey D. Martins M. B. Machado D. E.. (2018). Fc receptor-like 3 (-169TC) polymorphism increases the risk of tendinopathy in volleyball athletes: a case control study. BMC Med. Genet. 19:119. 10.1186/s12881-018-0633-6 30021560 PMC6052601

[B64] September A. V. Cook J. Handley C. J. van der Merwe L. Schwellnus M. P. Collins M. (2009). Variants within the COL5A1 gene are associated with Achilles tendinopathy in two populations. Br. J. Sports Med. 43, 357–365. 10.1136/bjsm.2008.048793 18443036

[B65] Shukla M. Gupta R. Pandey V. Tiwari P. K. Amrathlal R. S. (2020). COLIA1 + 1245 G > T Sp1 binding site polymorphism is not associated with ACL injury risks among indian athletes. Indian J Orthop. 54, 647–654. 10.1007/s43465-020-00119-1 32850029 PMC7429625

[B66] Somerville R. P. T. Oblander S. A. Apte S. S. (2003). Matrix metalloproteinases: old dogs with new tricks. Genome Biol. 4:216. 10.1186/gb-2003-4-6-216 12801404 PMC193609

[B67] Speakman J. R. (2015). The ‘fat mass and obesity related' (FTO) gene: mechanisms of impact on obesity and energy balance. Curr. Obes. Rep. 4, 73–91. 10.1007/s13679-015-0143-1 26627093

[B68] Spurway N. C. (1992). Aerobic exercise, anaerobic exercise and the lactate threshold. Br Med Bull. 48, 569–591. 10.1093/oxfordjournals.bmb.a072564 1450885

[B69] Stepien-Słodkowska M. Ficek K. Eider J. Leońska-Duniec A. Maciejewska-Karłowska A. Sawczuk M.. (2013). The +1245g/t polymorphisms in the collagen type I alpha 1 (col1a1) gene in polish skiers with anterior cruciate ligament injury. Biol. Sport 30, 57–60. 10.5604/20831862.1029823 24744467 PMC3944561

[B70] Tittel K. (2016). Beschreibende und Funktionelle Anatomie, 16. Überarbeitete und Erweiterte Auflage. München: Kiener.

[B71] Voisin S. Guilherme João Paulo F. L. Yan X. Pushkarev V. P. Cieszczyk P. Massidda M.. (2016). ACVR1B rs2854464 Is associated with sprint/power athletic status in a large cohort of europeans but not Brazilians. PLoS ONE 11:e0156316. 10.1371/journal.pone.0156316 27253421 PMC4890799

[B72] Wang P. Fedoruk M. N. Rupert J. L. (2008). Keeping pace with ACE: are ACE inhibitors and angiotensin II type 1 receptor antagonists potential doping agents? Sports Med. 38, 1065–1079. 10.2165/00007256-200838120-00008 19026021

[B73] Windelinckx A. Mars G. de Huygens W. Peeters M. W. Vincent B. Wijmenga C.. (2011). Comprehensive fine mapping of chr12q12-14 and follow-up replication identify activin receptor 1B (ACVR1B) as a muscle strength gene. Eur. J. Hum. Genet. 19, 208–215. 10.1038/ejhg.2010.173 21063444 PMC3025799

[B74] Yang N. Macarthur D. G. Gulbin J. P. Hahn A. G. Beggs A. H. Easteal S.. (2003). ACTN3 genotype is associated with human elite athletic performance. Am. J. Hum. Genet. 73, 627–631. 10.1086/377590 12879365 PMC1180686

[B75] Yang R. Shen X. Wang Y. Voisin S. Cai G. Fu Y.. (2017). ACTN3 R577X gene variant is associated with muscle-related phenotypes in elite chinese sprint/power athletes. J. Strength Cond. Res. 31, 1107–1115. 10.1519/JSC.0000000000001558 27442335

[B76] Zarebska A. Jastrzebski Z. Ahmetov I. I. Zmijewski P. Cieszczyk P. Leonska-Duniec A.. (2017). GSTP1 c.313AG polymorphism in Russian and Polish athletes. Physiol. Genomics 49, 127–131. 10.1152/physiolgenomics.00014.2016 28062686

