# Supplementary material for: Effects of Genetic Variation on Endurance Performance, Muscle Strength, and Injury Susceptibility in Sports: A Systematic Review
Source: Front Physiol. 2021 Jul 21;12:694411. doi: 10.3389/fphys.2021.694411 (PMC8334364; doi:10.3389/fphys.2021.694411)
Supplement: Supplementary file 1 [file Table_1.DOCX]

Supplementary Material

# Supplementary Data

| **No.** | **Title** | **Author** | **Database** | **Year** |
| --- | --- | --- | --- | --- |
| **Topic:** genetic polymorphism for endurance & power, **Database:** PubMed, Web of Science; **Period:** 01.10. – 12.12.2019  **Search Term:** athlete* OR professional player* OR professional athlete* OR elite athlete status* OR athletic status* OR competitive player* OR elite professional player* OR top-level athlete* OR top level athlete* OR competitive athlete* AND genetic variant* OR sports relevant polymorphism* OR genetic influence* OR genetic biomarker* OR genetic marker* OR polymorphism* OR single nucleotide polymorphism* OR genetic polymorphism* AND endurance capacity* OR endurance performance* OR endurance exercise* OR endurance* OR physical strength* OR power performance* OR power output* OR power sports performance* OR muscle power* OR muscle strength* OR power exercise* | | | | |
| 1 | GSTP1 c.313A>G polymorphism in Russian and Polish athletes | Zarabska et al. [48] | PubMed | 2017 |
| 1a | GSTP1 c.313A>G polymorphism in Russian and Polish athletes | Zarabska et al. [48] | PubMed | 2017 |
| 2 | Genetic variants associated with physical and mental characteristics of the elite athletes in the Polish population | Peplonska et al. [45] | PubMed  Web of Science | 2017 |
| 3 | Association of ACE gene polymorphism with cardiovascular determinants of trained updates and untrained Iranian men | Falahati et al. [38] | Web of Science | 2019 |
| 4 | The A-allele of the FTO Gene rs9939609 polymorphism is associated with decreased proportion of slow oxidative muscle fibers and over-represented in heavier athletes | Guilherme et al. [40] | Web of Science | 2019 |
| 5 | ACTN3 genotype in professional sport climbers | Ginszt et al. [39] | Web of Science | 2018 |
| 6 | Single nucleotide polymorphisms in carnosinase genes (CNDP1 and CNDP2) are associated with power athletic status | Guilherme et al. [41] | Web of Science | 2017 |
| 7 | ACTN3 R577X gene variant is associated with muscle-related phenotypes in elite Chinese sprint/power athletes | Yang et al. [47] | Web of Science | 2017 |
| 8 | ACTN3 R577X genotype and performance of elite middle-long distance swimmers in China | Li et al. [44] | Web of Science | 2017 |
| 9 | The Association Between MCT1 T1470A Polymorphism and Power-Oriented Athletic Performance | Kikuchi et al. [43] | Web of Science | 2017 |
| 10 | ACVR1B rs2854464 is associated with sprint/ power athletic status in a large cohort of Europeans but not Brazilians | Voisin et al. [46] | Web of Science | 2016 |
| 11 | Is there a relationship between PPARD T294C/PPARGC1A Gly482Ser variations and physical endurance performance in the Korean population? | Jin et al. [42] | Web of Science | 2016 |
| 12 | IGF-1 receptor 275124 A>C (rs1464430) polymorphism and athletic performance | Ben-Zaken et al. [37] | Web of Science | 2015 |
| **Topic:** genetic polymorphism for susceptibility to injury; Database: PubMed, Web of Science; Period: 01.10. – 12.12.2019  **Search Term:** athlete* OR professional player* OR professional athlete* OR elite athlete status* OR athletic status* OR competitive player* OR elite professional player* OR top-level athlete* OR top level athlete* OR competitive athlete* AND genetic variant* OR sports relevant polymorphism* OR genetic influence* OR genetic biomarker* OR genetic marker* OR polymorphism* OR single nucleotide polymorphism* OR genetic polymorphism* AND susceptibility to injury* OR muscle stiffness* OR soft tissue injury* OR tendinopathy* OR injury risk* OR ligament rupture* OR muscle injury* OR muscle strain injury* OR muscle damage* OR musculoskeletal soft tissue injury* OR ligament injury* OR tendon injury* OR injury* | | | | |
| 13 | Are TNC gene variants associated with anterior cruciate ligament rupture susceptibilty | Lulińska-Kuklik et al. [51] | PubMed | 2019 |
| 14 | Fc receptor-like 3 (-169T>C) polymorphism increases the risk of tendinopathy in volleyball athletes: a case control study | Salles et al. [53] | PubMed  Web of Science | 2018 |
| 15 | BMP4 and FGF3 haplotypes increase the risk of tendinopathy in volleyball athletes | Salles et al. [54] | PubMed  Web of Science | 2015 |
| 16 | Interactions between COL5A1 gene and risk of the anterior cruciate ligament rupture | Lulińska-Kuklik et al. [52] | Web of Science | 2018 |
| **Topic**: genetic polymorphism for susceptibility to injury; Database: PubMed, Web of Science; Period: 01.10. – 12.12.2019  **Search Term:** ((elite athlete OR athlete) AND (genetic polymorphism OR genetic variant OR polymorphism OR single nucleotide polymorphism)) AND (susceptibility to injury OR injury risk OR soft tissue injury OR ligament rupture OR musculoskeletal soft tissue injury) | | | | |
| 17 | Are IL1B, IL6 and IL6R gene variants associated with anterior cruciate ligament rupture susceptibility | Lulińska-Kuklik et al. [49] | PubMed | 2019 |
| 18 | Are MMP3, MMP8 and TIMP2 gene variants associated with anterior cruciate ligament rupture susceptibility | Lulińska-Kuklik et al. [50] | PubMed | 2019 |

***Table S1.*** **Detailed search strategy including topic, searched databases, search period and exact search term.** Electronic databases were searched from October 2019 to the middle of December 2019. The table shows the studies included in the systematic review.

| **Bias Category** | **Question** | **High risk of bias** | **Low risk of bias** | **Unclear risk of bias** | **Evaluation** | **Notes** |
| --- | --- | --- | --- | --- | --- | --- |
| Selection Bias  Selection of participants | Has there been an adequate selection of study participants? | Selection bias due to an inadequate selection of study participants | Sensible selection of study participants | Not described in detail or unclear | High risk (↑)  Low risk (↓)  Unclear risk (?) | Were the groups clearly defined and homogenous? Is the CG suitably selected? Is there a representativeness for the target population? |
| Selection Bias  Disruptive factors | Are the main interference factors identified and adequately considered in the design and analysis? | No identification of the disruptive factors  and consideration of these in the analysis | Identification of disruptive factors and consideration of these in the analysis | Not described in detail or unclear | High risk (↑)  Low risk (↓)  Unclear risk (?) | Were appropriate procedures used to deal with disruptive factors? |
| Performance Bias  Definition of exposure | Is the exposure clearly defined? | Bias due to insufficient definition of exposure | Clear definition of exposure | Not described in detail or unclear | High risk (↑)  Low risk (↓)  Unclear risk (?) | - |
| Exposure measurement | Is the measurement method used to determine exposure appropriate? | Bias due to inappropriate application of the measurement method | Appropriate use of the measurement method | Not described in detail or unclear | High risk (↑)  Low risk (↓)  Unclear risk (?) | - |
| Detection Bias  Blinding of the result evaluation | Has the inspectors been blinded during the measurement of results? | Bias due to missing listing of measures for blinding or no evidence of blinding of the examiners during result measurement | Listing of measures for blinding or indications of blinding of the testers of the result measurement | Not described in detail or unclear | High risk (↑)  Low risk (↓)  Unclear risk (?) | Was reference made to blinding measures or was blinding explicitly mentioned in the text? |
| Reporting Bias  Selective reporting of results | Has all data been presented in full and if not, has the author(s) given a clear explanation for the lack of data? | Bias due to selective reporting of results and/or insufficient justification in case of non-disclosure of data | No selective reporting on results discernible | Not described in detail or unclear | High risk (↑)  Low risk (↓)  Unclear risk (?) | Were only significant or also non-significant results reported?  Is the display of data missing? |
| CG = control group | | | | | | |

***Table S 2*. Presentation and explanation of quality assessment using the RoBANS tool based on the Cochrane ROB tool [30,56,57]**
